# Cu_3_SbSe_3_‐Alloying‐Induced High Thermoelectric Performance and Mechanical Robustness in Bi_2_Te_3_‐Based Thermoelectric Materials

**DOI:** 10.1002/advs.202512417

**Published:** 2025-08-19

**Authors:** Ruiheng Li, Xiao‐Lei Shi, Jianglong Zhu, Qian Deng, Wenxin Ou, Jie Zheng, Xiaobo Tan, Xuri Rao, Qiang Sun, Min Hong, Ran Ang, Zhi‐Gang Chen

**Affiliations:** ^1^ Key Laboratory of Radiation Physics and Technology Ministry of Education Institute of Nuclear Science and Technology Sichuan University Chengdu 610064 China; ^2^ School of Chemistry and Physics ARC Research Hub in Zero‐emission Power Generation for Carbon Neutrality and Centre for Materials Science Queensland University of Technology Brisbane QLD 4000 Australia; ^3^ State Key Laboratory of Oral Diseases National Clinical Research Center for Oral Diseases West China Hospital of Stomatology Sichuan University Chengdu Sichuan 610041 China; ^4^ Centre for Future Materials University of Southern Queensland Springfield Campus Springfield Central QLD 4300 Australia; ^5^ Institute of New Energy and Low‐Carbon Technology Sichuan University Chengdu 610065 China; ^6^ College of Physics Sichuan University Chengdu 610064 China

**Keywords:** alloying, bismuth telluride, conversion efficiency, mechanical property, thermoelectric

## Abstract

Bi_2_Te_3_‐based thermoelectric materials remain the only commercially viable candidates for low‐grade waste heat recovery. However, their moderate thermoelectric performance and limited mechanical robustness constrain broader industrial applications. Here, a synergistic enhancement of both the thermoelectric and mechanical properties of Bi_0.4_Sb_1.6_Te_3.01_ is demonstrated by alloying with Cu_3_SbSe_3_ via high‑energy ball milling followed by hot pressing. This approach optimizes carrier concentration and reduces microscale porosity, yielding a significant improvement in the power factor across the entire temperature range. Simultaneously, the introduction of stacking faults and dislocations intensifies phonon scattering, effectively suppressing lattice thermal conductivity. As a result, the optimized sample achieves a peak *zT* of ≈1.45 at 378 K and an average *zT* of ≈1.3 over 303–503 K. Its mechanical properties are also substantially enhanced, with a Vickers hardness of 96 Hv and a compressive strength of 187 MPa. A 7‑pair thermoelectric device fabricated from the optimized material delivers a maximum conversion efficiency of ≈6.9% at a temperature difference of 182 K. This work highlights the efficacy of combining microstructural engineering with strategic alloying as a promising route to advance both the thermoelectric and mechanical performance of Bi_2_Te_3_‑based materials.

## Graphical abstract

Combining microstructural engineering with Cu_3_SbSe_3_ alloying significantly improves the performance of Bi_2_Te_3_‑based thermoelectric materials, yielding a high *zT* of ≈1.45 (average *zT* ≈1.3 over 303–503 K), excellent mechanical robustness (Vickers hardness of 96 Hv and compressive strength of 187 MPa), and an impressive conversion efficiency of ≈6.9% at a Δ*T* of 182 K.

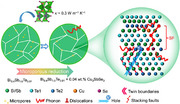

## 1 Introduction

The efficiency of internal combustion engines is intrinsically limited, with roughly 60% of fossil fuel energy lost as waste heat.^[^ ^1^ ^]^ Harvesting and converting this waste thermal energy into electricity presents a compelling approach for addressing the global energy crisis.^[^ ^2^ ^]^ Thermoelectric generators have gained significant attention due to their ability to directly and reliably convert thermal energy into electrical power in an environmentally friendly manner.^[^ ^3^ ^]^ However, their widespread implementation is severely constrained by the inherent properties of thermoelectric materials. The performance of thermoelectric materials is evaluated by the dimensionless figure of merit, *zT* = *S* ^2^ *σT*/*κ*, where *σ* is the electrical conductivity, *S* is the Seebeck coefficient, *T* is the absolute temperature, and *κ* is the total thermal conductivity, consisting of lattice (*κ*_l_), electronic (*κ*_e_), and bipolar (*κ*_b_) contributions.^[^ ^4^ ^]^ Higher *zT* values enable greater conversion efficiency. To this end, various strategies have been developed to optimize *zT*, including carrier concentration tuning, band structure engineering, and phonon engineering through the design of multi‐scale microstructural features.^[^ ^5^ ^]^

Although several emerging thermoelectric materials, such as Ag_2_Se^[^ ^6^ ^]^ and Mg_3_(Sb, Bi)_2_,^[^ ^7^ ^]^ have drawn significant attention due to their near‐room‐temperature performance and broad application prospects, Bi_2_Te_3_‐based alloys remain the only commercially viable thermoelectric materials for room‐temperature applications.^[^ ^8^ ^]^ As typical layered compounds, Bi_2_Te_3_ alloys feature weak van der Waals bonding between layers, making them prone to dissociation along the *c*‐axis and resulting in high mechanical fragility and limited processing capability.^[^ ^9^ ^]^ To enable large‐scale commercialization of Bi_2_Te_3_‐based thermoelectric materials and devices, it is critical to optimize both their thermoelectric and mechanical properties.^[^ ^8^, ^10^ ^]^ In particular, robust mechanical strength is vital for long‐term stability and reliability under the thermal and mechanical stresses encountered in service.^[^ ^11^ ^]^ Moreover, with Bi_2_Te_3_‐based modules increasingly being explored for advanced applications such as human‐machine interfaces, biomedical devices, and nuclear energy recovery, there is an urgent need for materials with enhanced mechanical properties that can satisfy the stringent requirements of these emerging fields.^[^ ^12^ ^]^

In *p*‐type Bi_2_Te_3_, excess Te is commonly introduced to optimize thermoelectric performance. For example, liquid‐phase compaction with ≈25 wt.% Te promotes the formation of intensive dislocation arrays at low‐energy grain boundaries, effectively scattering intermediate‐frequency phonons and significantly reducing *κ*_l_.^[^ ^13^ ^]^ Meanwhile, the addition of 2–5 wt.% Te has been shown to lower the hole carrier concentration (*p*_H_) by suppressing the formation of anti‐site defect 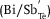, thereby enhancing electrical properties and reducing *κ*_e_.^[^ ^14^ ^]^ Similarly, trace doping with ≈1 mol% Te (Bi_0.4_Sb_1.6_Te_3.01_) can fine‐tune the defect chemistry by balancing the “donor‐like” effect (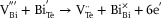) and mitigating 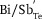 defects, yielding improved thermoelectric performance.^[^ ^15^ ^]^ However, this enhancement of thermoelectric properties often compromises mechanical strength, as trace amounts of Te segregating to grain boundaries can promote grain boundary sliding, while the volatilization of Te‐rich phases during sintering may introduce micro‐porosity.^[^ ^8d^ ^]^ Recently, dopants such as MgB_2_, Ag_8_GeTe_6_, Ag_5_SbSe_4_, and NaBiS_2_ have been shown to simultaneously enhance both thermoelectric and mechanical properties in *p*‐type Bi_2_Te_3_‐based materials.^[^ ^8^, ^11^, ^16^ ^]^ This solid‐solution strengthening approach not only optimizes *p*_H_ but also introduces a range of defect structures, including twin boundaries (TB) and dislocations. Notably, stacking faults (SF) often arise near grain boundaries, greatly increasing the likelihood of TB formation.^[^ ^12a^ ^]^ The resulting coherent and semi‐coherent TB structures effectively scatter phonons while preserving favorable charge transport properties.^[^ ^10^, ^17^ ^]^ Moreover, these TBs impede dislocation motion and can even absorb dislocations through interfacial migration, thereby simultaneously boosting the strength and plasticity of the material.^[^ ^10a^ ^]^ This strategy offers a promising pathway for overcoming the mechanical limitations of Bi_0.4_Sb_1.6_Te_3.01_ while further enhancing its thermoelectric performance.

**Figure 1:**
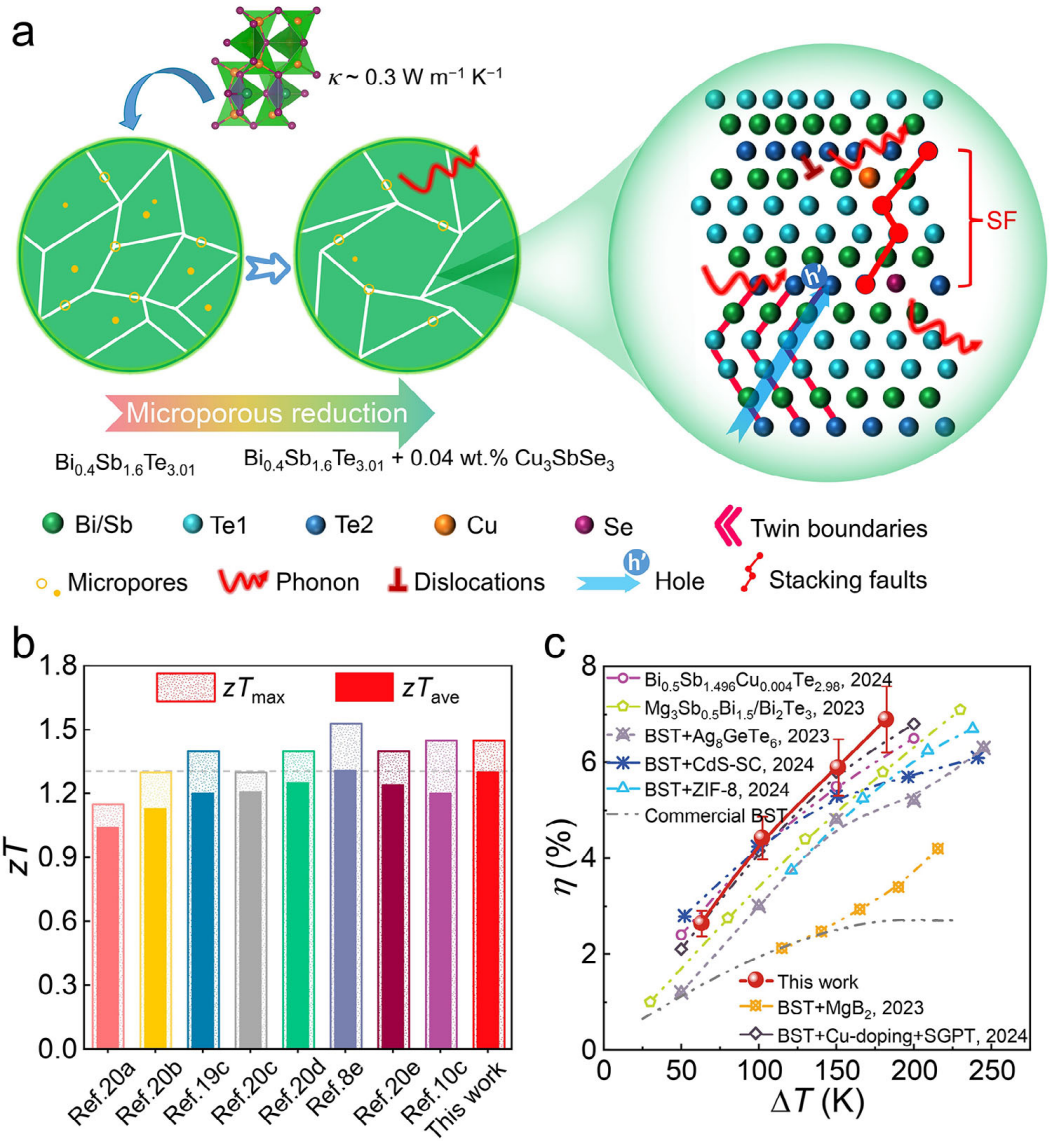
Introduction of the strategy for achieving high thermoelectric performance in *p*‐type Bi_2_Te_3_‐based thermoelectrics. a) Schematic illustration showing that incorporating the low‐thermal‐conductivity Cu_3_SbSe_3_ second phase into the Bi_0.4_Sb_1.6_Te_3.01_ matrix introduces various defects that more effectively scatter phonons, thereby reducing the total thermal conductivity (*κ*). Here, SF is abbreviated from stacking fault. b) Maximum and average *zT* (*zT*_max_ and *zT*_ave_) values achieved in this work, along with a comparison to results from similar studies.^[^ ^8^, ^10^, ^19^, ^20^ ^]^ c) Maximum conversion efficiency (*η*_max_) of a thermoelectric device based on this material across different temperature differences (Δ*T*) values, compared with those reported in the literature.^[^ ^8^, ^10^, ^11^, ^19^ ^]^ Here, BST is abbreviated from bismuth‐antimony‐telluride‐based materials, SC is abbreviated from super‐tetrahedron cluster, and ZIF is abbreviated from zeolitic imidazolate frameworks.

In this study, we simultaneously enhanced the thermoelectric and mechanical properties of Bi_0.4_Sb_1.6_Te_3.01_ by alloying it with the ternary, non‐stoichiometric compound Cu_3_SbSe_3_, which has an intrinsically low *κ* (≈0.3 W m^−1^ K^−1^) near room temperature.^[^ ^18^ ^]^ High‐energy ball (HBM) milling ensured the uniform dispersion of Cu_3_SbSe_3_ within the matrix lattice, which in turn facilitated the formation of 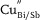 defects, thereby effectively optimizing the *p*_H_. Rapid hot pressing (HP) preserved this homogeneity during densification. Simultaneously, the reduction of microscale porosity and the introduction of coherent TB minimized the loss of carrier mobility (*µ*_H_), yielding a significant boost in the power factor (*PF*). Moreover, the formation of SFs and dislocations introduced strong phonon scattering, maintaining low *κ*, as illustrated in **Figure** **1**a. As a result, the optimized sample achieved a maximum figure of merit (*zT*_max_) of ≈1.45 at 378 K and an average zT (*zT*_ave_) of ≈1.3 over the range of 303–503 K (Figure 1b). These microstructural refinements also strengthened the material, yielding a Vickers hardness of 96 Hv and a compressive strength of 187 MPa. A 1 cm × 1 cm thermoelectric device, composed of this *p*‐type sample and an *n*‐type hot‐extruded Bi_2_Te_2.7_Se_0.3_ leg, demonstrated a maximum conversion efficiency (*η*_max_) of ≈6.9% under a temperature gradient (Δ*T*) of 182 K (Figure 1c).^[^ ^8^, ^10^, ^11^, ^19^ ^]^ This work highlights the effectiveness of combining microstructural engineering and targeted doping strategies to simultaneously optimize the thermoelectric and mechanical performance of Bi_2_Te_3_‐based materials.

## 2 Results and Discussion

**Figure 2:**
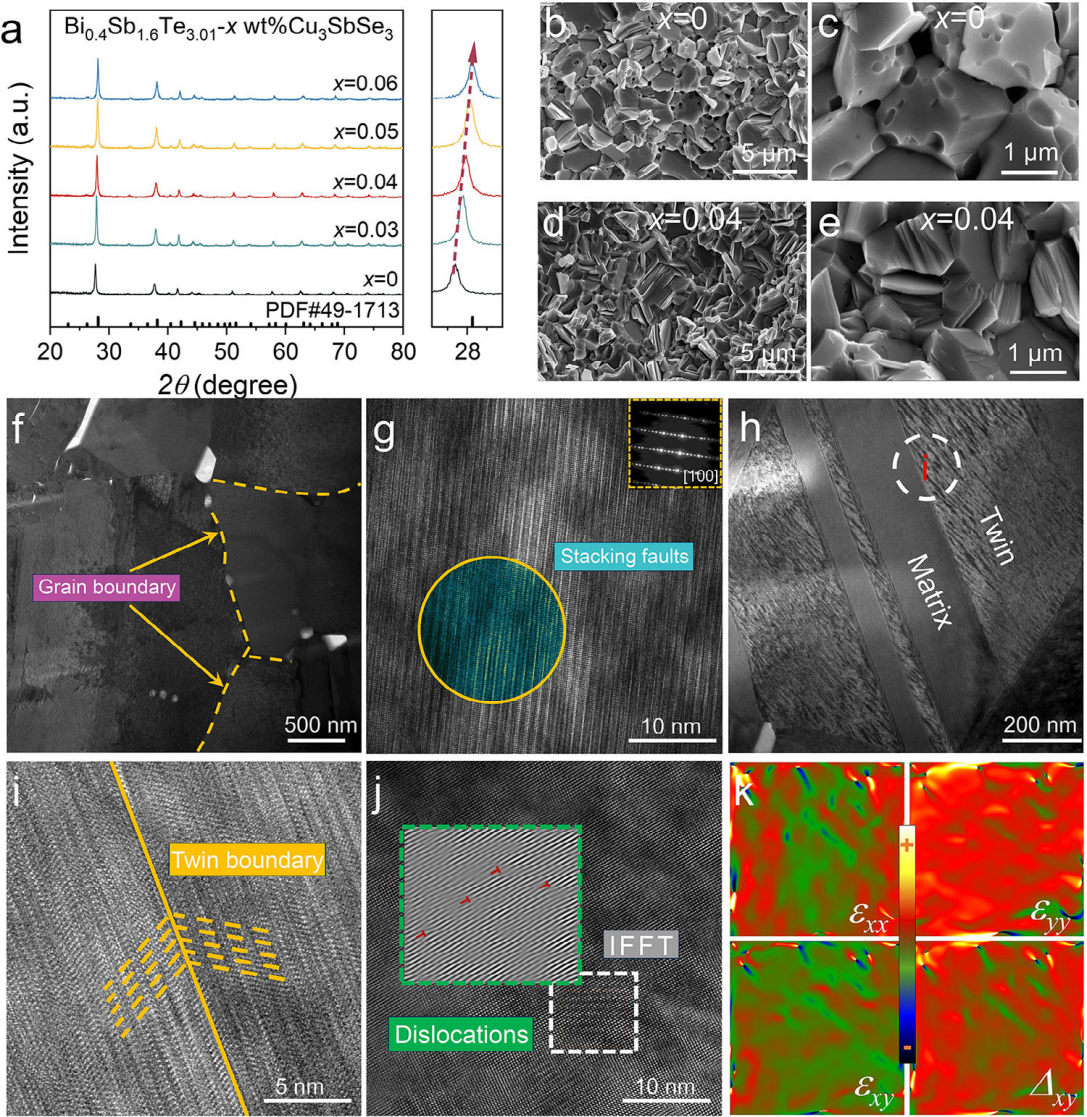
Characterizations of Bi_0.4_Sb_1.6_Te_3.01_ + 0.04 wt.% Cu_3_SbSe_3_ sample. a) X‐ray diffraction (XRD) patterns of Bi_0.4_Sb_1.6_Te_3.01_ + *x* wt.%Cu_3_SbSe_3_ samples. b,c) Fracture surface image and magnified view of Bi_0.4_Sb_1.6_Te_3.01_. d,e) Fracture surface image and magnified view of Bi_0.4_Sb_1.6_Te_3.01_ + 0.04 wt.% Cu_3_SbSe_3_. f) Low‐magnification transmission electron microscopy (TEM) image showing micropores and grain boundaries. g) High‐resolution TEM (HRTEM) image of high‐intensity stacking faults. h,i) Low‐magnification TEM image displaying twin boundaries (TB) and HRTEM of localized regions of "i". j) Inverted fast Fourier transform (IFFT) images of selected regions highlighting dislocations. k) Geometric phase analysis (GPA) illustrating the stress–strain distribution.

As shown in **Figure** **2**a, the phase structure of Bi_0.4_Sb_1.6_Te_3.01_ + *x *wt.% Cu_3_SbSe_3_ samples were characterized by X‐ray diffraction (XRD) (The phase structure of the pre‐synthesized Cu_3_SbSe_3_ is shown in Figure S1, Supporting Information). All diffraction peaks can be well‐indexed to the standard Bi–Sb–Te pattern (PDF#49‐1713) with a space group of $R\bar{3}m$.^[^ ^21^ ^]^ In the enlarged XRD profiles near 28°, the primary diffraction peak shifts systematically toward higher angles with increasing Cu_3_SbSe_3_ content, indicating a contraction of the lattice parameters. This shift is likely attributable to the atomic size differences between Cu (117 pm), Bi (146 pm), Sb (141 pm), Te (137 pm), and Se (117 pm). In addition, energy‐dispersive X‐ray spectroscopy (EDS) elemental mapping (Figure S2, Supporting Information) confirms the uniform distribution of all constituent elements throughout the samples.

To assess the influence of Cu_3_SbSe_3_ doping on the microstructure of Bi_0.4_Sb_1.6_Te_3.01_, the fracture morphology and elemental distribution of Bi_0.4_Sb_1.6_Te_3.01_ + 0.04 wt.% Cu_3_SbSe_3_ were examined by SEM. Low‑magnification images (Figure S3a,b, Supporting Information) reveal the overall microstructure, with grain size statistics (via Gaussian fitting) indicating negligible changes upon doping (Figure S3c,d, Supporting Information). Figure 2b–e presents the fracture surface image and magnified views of Bi_0.4_Sb_1.6_Te_3.01_ and Bi_0.4_Sb_1.6_Te_3.01_ + 0.04 wt.% Cu_3_SbSe_3_, respectively. All samples display characteristic transgranular fracture surfaces and scattered micropores, presumably arising from the volatilization of the Te‑rich phase. Due to the low amount of excess Te, these micropores have a negligible effect on sample density, as shown in Table S1 (Supporting Information). In Bi_0.4_Sb_1.6_Te_3.01_, the micropores occur both within the grains and at grain boundaries, whereas in Bi_0.4_Sb_1.6_Te_3.01_ + 0.04 wt.% Cu_3_SbSe_3_, they are primarily located along the grain boundaries. This shift may be attributed to the defect redistribution and Sb incorporation at cation vacancy sites, which help balance the cation‐to‐anion ratio and suppress excessive pore formation. The grain‑boundary pores mitigate carrier scattering while providing phonon scattering, thereby maintaining low *κ*. Moreover, the reduced pore intensity alleviates stress concentration, resulting in improved strength and hardness of the material.

Micro‐ and nanostructures play a pivotal role in determining thermoelectric properties. To investigate the microstructural evolution at the nanoscale, Bi_0.4_Sb_1.6_Te_3.01_ + 0.04 wt.% Cu_3_SbSe_3_ samples were analyzed by transmission electron microscopy (TEM). As shown in Figure S4 (Supporting Information), Bi, Sb, Cu, Te, and Se are uniformly distributed, confirming the successful incorporation of Cu_3_SbSe_3_ into the Bi_0.4_Sb_1.6_Te_3.01_ matrix. Moreover, based on the (*χ*, *r*) model,^[^ ^15d^ ^]^ Se is prone to occupy Te‐site substitutional defects. Figure 2f highlights grain boundaries (marked by yellow dashed lines) and nanoscale pores primarily located along these boundaries. The high‐resolution TEM (HRTEM) image (Figure 2g) reveals the presence of SFs under HBM, with the inset showing the selected‐area electron diffraction (SAED) pattern, which confirms that the SF lies on the [100] basal plane. Moderate SF intensities serve as effective barriers to dislocation motion, thereby enhancing the material strength and hardness. TBs were identified in low‐magnification TEM images, as illustrated in Figure 2h. The formation of TBs is attributed to the low SF energy of the Bi_0.4_Sb_1.6_Te_3.01_ + 0.04 wt.% Cu_3_SbSe_3_ sample, with SFs near grain boundaries facilitating TB generation. Compared to adjacent regions, the TB area exhibited a sharp contrast from the surrounding matrix. This marked region “i” in Figure 2h was selected for further analysis by HRTEM, as shown in Figure 2i, which confirms that the lattice structure across the TB is highly symmetric and coherent, allowing for effective phonon scattering while preserving excellent charge transport properties. TBs, as special grain boundaries with low interfacial energies and high symmetry, are more stable than typical grain boundaries, providing an effective barrier to dislocation motion and thereby further increasing the strength and hardness of the material. Figure 2j shows an area with high‐intensity atomic‐scale dislocations revealed by inverse fast Fourier transform (IFFT) analysis, and Figure 2k presents the associated strain fluctuations observed by geometric phase analysis (GPA), highlighting the direct correlation between lattice defects and strain distribution.

**Figure 3:**
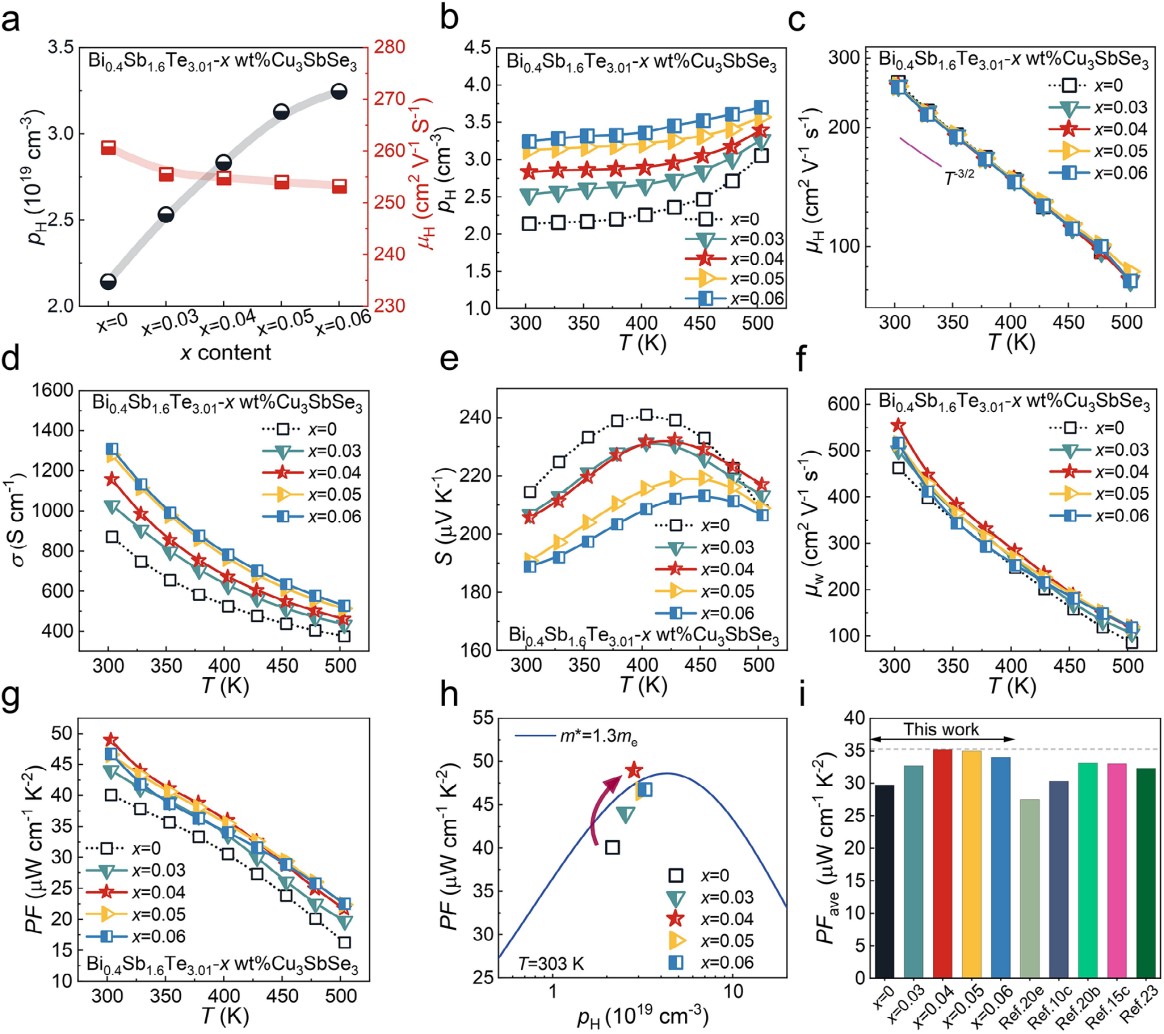
Electrical transport properties of Bi_0.4_Sb_1.6_Te_3.01_ + *x* wt.% Cu_3_SbSe_3_ samples. a) Carrier transport properties at 303K. Temperature‐dependent properties: b) hole carrier concentration (*p*_H_), c) hole carrier mobility (*µ*_H_), d) electrical conductivity (*σ*), e) Seebeck coefficient (*S*), f) weight mobility (*µ*_w_), and g) power factor (*PF = S* ^2^ *σ*). h) Relationship between *p*_H_ and *PF* at 303 K. i) Comparison of the average *PF* (*PF*_ave_) of this work with previous reports.^[^ ^10^, ^15^, ^20^, ^23^ ^]^

To elucidate the influence of Cu_3_SbSe_3_ addition on the charge transport behavior, **Figure** **3**a summarizes the charge transport properties of Bi_0.4_Sb_1.6_Te_3.01_ + *x* wt.% Cu_3_SbSe_3_ at 303 K, highlighting the variations in *σ* and *S*. As the Cu_3_SbSe_3_ content increases, the *p*_H_ rises, primarily due to the contribution of 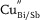 in generating additional hole carriers. At 503 K (Figure 3b), *p*_H_ further increases due to the thermal excitation of carriers.^[^ ^22^ ^]^ In addition, the onset temperature for intrinsic excitation shifts from 378 to 403 K, suggesting a potential widening of the bandgap. The temperature dependence of the *µ*_H_ for all samples follows a *T* ^−3/2^ trend, indicating that charge transport is dominated by acoustic phonon scattering (Figure 3c).^[^ ^20e^ ^]^ Notably, *µ*_H_ shows only a slight decrease when *x* ≥ 0.03, which can be attributed to the reduced micropore intensity and the presence of coherent twin boundaries that help mitigate carrier scattering.

In Figure 3d, the *σ* decreases monotonically with increasing temperature, a characteristic behavior of degenerate semiconductors. The observed rise in *σ* upon the addition of Cu_3_SbSe_3_ can be attributed to the enhanced *p*_H_. Figure 3e shows the *S*, where the positive sign confirms *p*‐type conduction. *S* initially increases with temperature but subsequently decreases due to thermal excitation of minority carriers, indicative of the bipolar conduction effect.^[^ ^4a^ ^]^ Notably, the temperature at which *S* reaches its maximum shifts to higher temperatures with increasing Cu_3_SbSe_3_ content, suggesting an effective suppression of the bipolar effect.

The charge carrier transport properties were analyzed using an effective‐mass model, with detailed calculations presented in the Supporting Information. The Pisarenko plots (*p*_H_–*S*) at 303 K, shown in Figure S5 (Supporting Information), reveal that the density‐of‐states effective mass (*m* ^*^) is ≈1.3 m_e_ across all Bi_0.4_Sb_1.6_Te_3.01_ + *x* wt.% Cu_3_SbSe_3_ samples, indicating that the band structure remains largely unaffected by Cu_3_SbSe_3_ doping. Density functional theory (DFT) calculations for Bi_0.4_Sb_1.6_Te_3.01_ and Bi_0.4_Sb_1.6_Te_3.01_ + 0.04 wt.% Cu_3_SbSe_3_ further confirm this trend, demonstrating that Cu_3_SbSe_3_ has minimal impact on the overall band structure (Figure S6, Supporting Information). However, the calculations reveal a slight widening of the bandgap and a shift of the Fermi level deeper into the valence band upon Cu_3_SbSe_3_ incorporation, supporting its role in suppressing the bipolar diffusion effect and increasing *p*_H_. The electron localization functions (ELFs) for Bi_0.4_Sb_1.6_Te_3.01_ and Bi_0.4_Sb_1.6_Te_3.01_ + 0.04 wt.% Cu_3_SbSe_3_ are shown in Figure S7 (Supporting Information), with the corresponding crystal structures presented in Figure S8 (Supporting Information). The ELF scale ranges from 0 to 1, where lower values (blue) denote off‐domain electron localization. In the Cu_3_SbSe_3_‐doped sample, off‐domain regions (ELF ≈0) appear prominently as blue circular domains around Cu atoms, alongside slightly green domains near Se atoms, indicating significant electron delocalization. This explains the minimal reduction in *µ*_H_ observed for the *x* = 0.04 composition.

The negligible decrease in *µ*_H_ allows *S* to remain at a high level, thereby enhancing the weighted mobility (*µ*_w_) across all doped samples (Figure 3f), indicating improved charge transport characteristics. As a result, the combined effect of increased *σ* and preserved high *S* leads to a significant improvement in the *PF* (Figure 3g). Notably, the *PF* increased from 40 µW cm^−1^ K^−2^ for pristine Bi_0.4_Sb_1.6_Te_3.01_ to 49 µW cm^−1^ K^−2^ for the *x* = 0.04 sample. Figure 3h illustrates the *PF* as a function of *p*_H_, showing a shift from the gray dashed line toward the blue solid line, approaching the optimal *p*_H_ range required for maximum *PF* (*PF*_max_). As shown in Figure 3i, the average *PF* (*PF*_ave_) of the *x* = 0.04 sample between 303 and 503 K reaches 35.2 µW cm^−1^ K^−2^, surpassing those of Bi_2_Te_3_‐based materials doped with other Cu‐based compounds.^[^ ^10^, ^15^, ^20^, ^23^ ^]^ This high *PF*_ave_ is advantageous for achieving an improved *zT*_ave_ and higher thermoelectric module output power.

**Figure 4:**
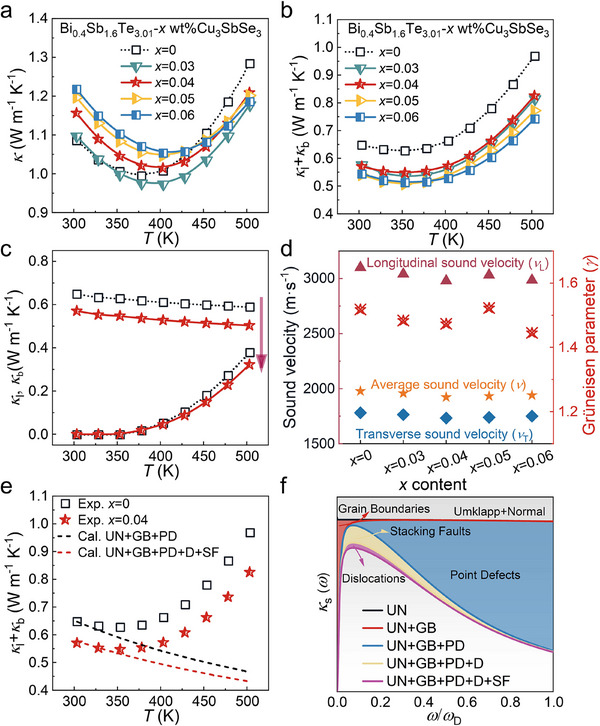
Thermal transport properties of Bi_0.4_Sb_1.6_Te_3.01_ + *x* wt.% Cu_3_SbSe_3_ samples. Temperature‐dependent a) thermal conductivity (*κ*), b) lattice thermal conductivity (*κ*_l_) + bipolar thermal conductivity (*κ*_b_)_,_ and c) *κ*_l_ and *κ*_b_. d) Room temperature sound velocities: longitudinal (*v*_L_), transverse (*v*_T_), and average (*v*) velocities, along with the Grüneisen parameter (*γ*). e) Experimental and calculated *κ*_l_ based on the Debye–Callaway model (without considering bipolar diffusion). f) Frequency‐dependent spectral lattice thermal conductivity (*κ*_s_) of Bi_0.4_Sb_1.6_Te_3.01_. Here, UN, GB, PD, D, and SF are abbreviated from Umklapp and Normal processes, grain boundaries, point defects, dislocations, and stacking faults, respectively.

In terms of thermal transport properties, **Figure** **4** illustrates the behavior of Bi_0.4_Sb_1.6_Te_3.01_ + *x* wt.% Cu_3_SbSe_3_. The thermal diffusivity (*D*) values are presented in Figure S9a (Supporting Information), and the *κ* value was calculated using the equation *κ* = *D* × *C*_p_ × *ρ*, where *C*_p_ (specific heat) was estimated based on the Dulong–Petit law and *ρ* is the geometrical density of the sample, determined by the Archimedes’ method. As shown in Figure 4a, the *κ* initially decreases to a minimum with increasing temperature and then rises due to the onset of bipolar conduction. Additionally, *κ* increases slightly with Cu_3_SbSe_3_ alloying. The *κ*_e_ was calculated for all samples using the Wiedemann–Franz law (Figure S9b, Supporting Information).^[^ ^24^ ^]^ By subtracting *κ*_e_ from the *κ*, the *κ*_l_ + *κ*_b_ were extracted (Figure 4b). The introduction of Cu_3_SbSe_3_ significantly suppresses *κ*_l_ + *κ*_b_. At room temperature, *κ*_l_ + *κ*_b_ decreased from 0.65 W m^−1^ K^−1^ in pristine Bi_0.4_Sb_1.6_Te_3.01_ to 0.54 W m^−1^ K^−1^ in the sample with 0.06 wt.% Cu_3_SbSe_3_. Since *κ*_b_ is negligible at low temperatures, *κ*_l_ is assumed to dominate, following an approximate 1/*T* behavior above the Debye temperature. Hence, *κ*_l_ values prior to the onset of bipolar conduction were estimated (Table S2, Supporting Information).^[^ ^25^ ^]^ As illustrated in Figure 4c, Cu_3_SbSe_3_ doping effectively suppresses both *κ*_l_ and *κ*_b_.

The sound velocities and Grüneisen parameters (*γ*) remained nearly unchanged with increasing Cu_3_SbSe_3_ content, indicating negligible lattice softening and minimal enhancement of lattice anharmonicity (Figure 4d). To evaluate the relative contributions of different scattering mechanisms in the microstructurally engineered samples, the Debye–Callaway model was applied, as shown in Figure 4e. The dominant phonon scattering mechanisms comprise Umklapp and Normal processes (UN), point defects (PD), grain boundaries (GB), SF, and dislocations (D). The gradual shift from the black to the red dashed lines reflects the effective *κ*_l_ reduction caused by SF and D. Figure 4f presents the frequency‐dependent spectral lattice thermal conductivity (*κ*_s_), where the reduced area between adjacent curves confirms the introduction of additional phonon scattering centers. UN and PD predominantly scatter high‐frequency phonons, while GB, SF, and D target low‐to‐mid‐frequency phonons. As a result, the rich variety of scattering centers promotes broad‐spectrum phonon scattering, yielding an effective reduction in *κ*_l_.

**Figure 5:**
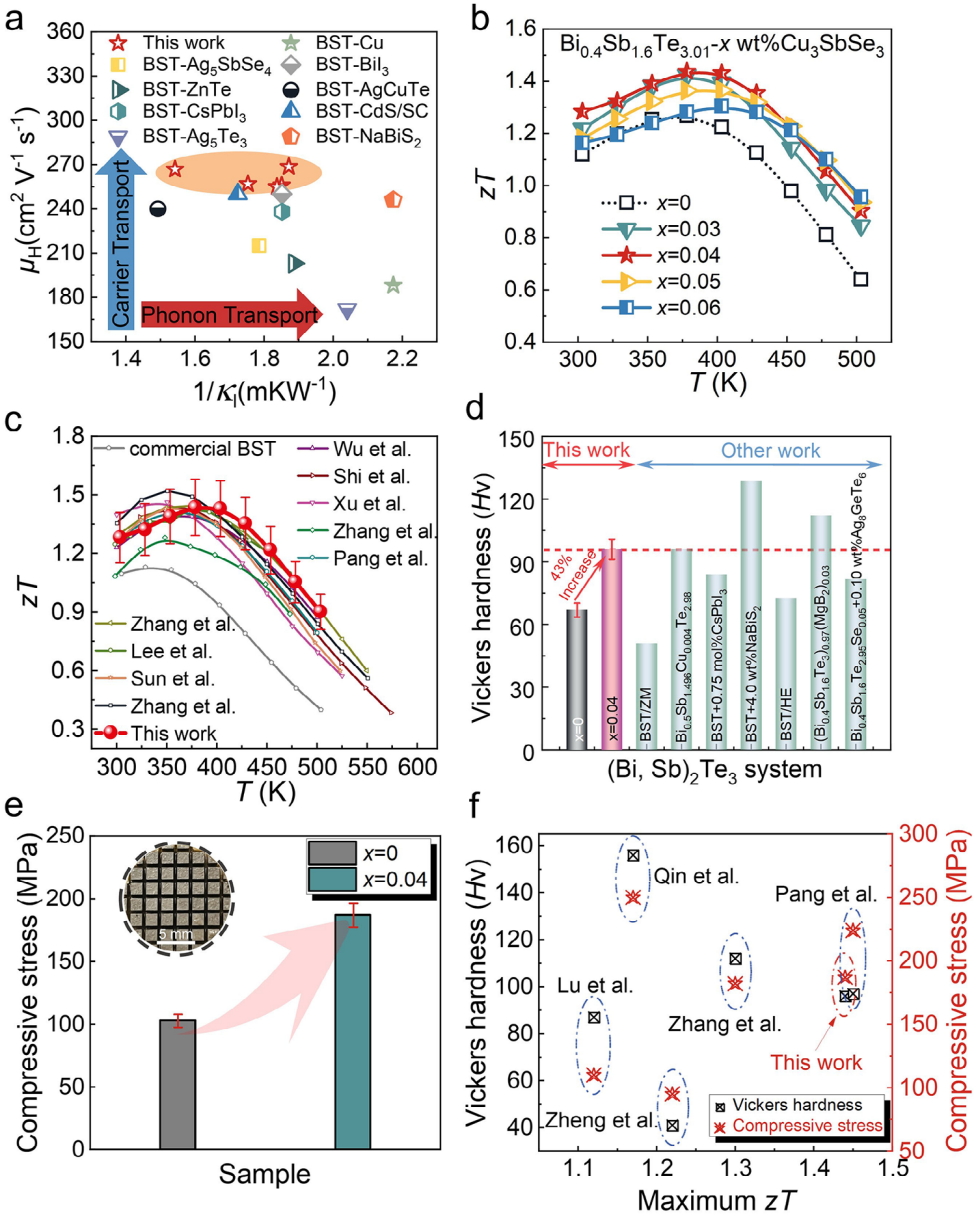
Thermoelectric and mechanical properties. a) Comparison of the degree of electroacoustic decoupling in this study with previously reported data.^[^ ^16^, ^19^, ^20^, ^26^ ^]^ b) Temperature‐dependent *zT*. c) Comparison of *zT* in this study with those reported for high‐performance *p*‐type (Bi, Sb)_2_Te_3_ alloys.^[^ ^8^, ^11^, ^16^, ^19^, ^23^, ^26^, ^27^ ^]^ d) Room‐temperature Vickers hardness compared with literature data.^[^ ^8^, ^10^, ^11^, ^16^, ^26^, ^29^ ^]^ e) Performance in the compressive stress test. f) Comparison of the maximum *zT*, Vickers hardness, and compressive stress of the *x* = 0.04 sample with some reports.^[^ ^10^, ^11^, ^30^ ^]^

Superior thermoelectric materials typically require a favorable combination of high *µ*_H_ and low *κ*_l_. **Figure** **5**a presents the inverse *κ*_l_ versus *µ*_H_, illustrating that the samples in this work outperform most recently reported (Bi, Sb)_2_Te_3_ alloys, indicating that doping effectively suppresses electron–phonon coupling.^[^ ^16^, ^19^, ^20^, ^26^ ^]^ As shown in Figure 5b, this leads to a significant enhancement in thermoelectric performance, with the *zT*_max_ rising from 1.26 for Bi_0.4_Sb_1.6_Te_3.01_ to ≈1.45 for the *x* = 0.04 sample at 378 K. The repeatability of the thermoelectric performance is confirmed in Figure S10 (Supporting Information). The anisotropy of (Bi, Sb)_2_Te_3_ was further evaluated by comparing transport properties along different orientations (Figure S11, Supporting Information), with the direction parallel to the hot‐pressing axis yielding superior performance. In addition to the improvement in *zT*_max_, the *zT* value is enhanced across the entire temperature range, resulting in a substantially higher *zT*_ave_. Figures 5c,1b compare the *zT* and *zT*_ave_ of the *x* = 0.04 sample with recent reports.^[^ ^8^, ^11^, ^16^, ^19^, ^23^, ^26^, ^27^ ^]^ For the *x* = 0.04 sample, a *zT*_ave_ of ≈1.3 was achieved over the temperature range of 303–503 K, which is highly competitive in Bi_2_Te_3_‐based system.^[^ ^28^ ^]^

To assess the mechanical performance, room‐temperature Vickers microhardness and uniaxial compression tests were conducted, as shown in Figure 5d,e, respectively. The introduction of Cu_3_SbSe_3_ significantly increased the Vickers hardness, from 67 Hv for Bi_0.4_Sb_1.6_Te_3.01_ to 96 Hv for the *x* = 0.04 sample, making it highly competitive compared with other reported (Bi, Sb)_2_Te_3_‐based materials (Figure 5d; Figure S12, Supporting Information).^[^ ^8^, ^10^, ^11^, ^16^, ^26^, ^29^ ^]^ The compressive stress–strain results (Figure 5e) further confirm that the *x* = 0.04 sample achieved a compressive strength of 187 MPa, making it well‐suited for subsequent mechanical processing and dicing for device assembly, as illustrated in the inset of Figure 5e. This remarkable strength is attributed to two main factors: 1) the reduction of micropore density, which minimizes stress concentrations, and 2) the introduction of high‐intensity SFs and TBs, which impede dislocation motion, prevent dislocation accumulation, and enable dislocation absorption and migration across interfaces, thereby enhancing the material plasticity. In the field of metals, the engineering of nanoscale microstructural features has long been recognized as an effective approach for achieving an optimal balance between superior mechanical and physical properties. These advances further improve the reliability and durability of thermoelectric modules. Figure 5f summarizes the *zT*_max_ and mechanical properties (Vickers hardness and compressive strength) of the *x* = 0.04 sample in comparison with other state‐of‐the‐art *p*‐type Bi_2_Te_3_‐based materials, highlighting its competitive advantage and underscoring its significance for practical thermoelectric applications.^[^ ^10^, ^11^, ^30^ ^]^

**Figure 6:**
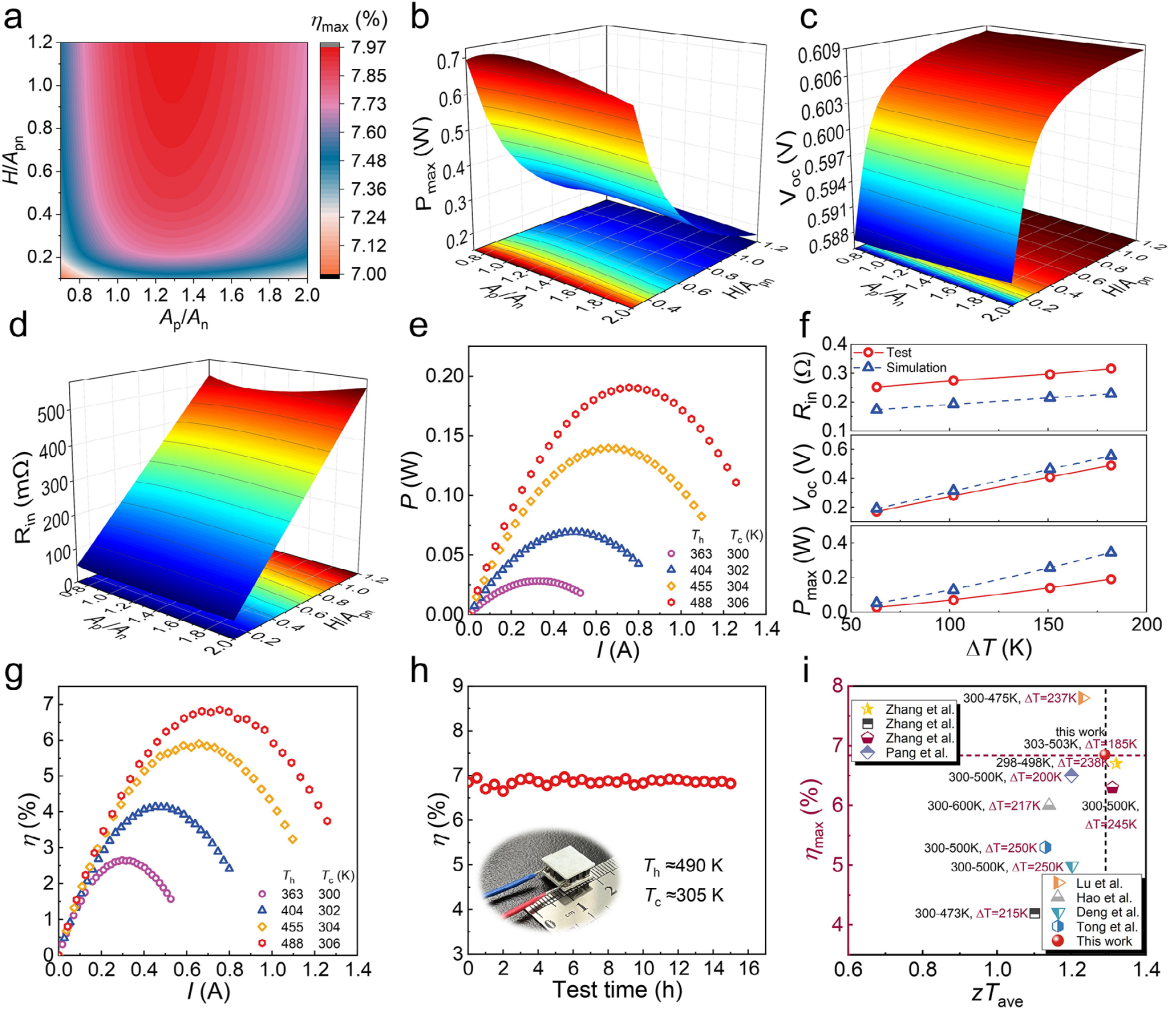
Simulation and tested results of the thermoelectric device based on Bi_0.4_Sb_1.6_Te_3.01_ + 0.04 wt.% Cu_3_SbSe_3_ bulks. a) Calculated 3D map projections of the *η*_max_ as a function of *H*/*A*_pn_ and *A*_p_/*A*_n_, where *H*, *A*_p_, and *A*_n_ are their leg height and the cross‐sectional areas of the *p*‐ and *n*‐type legs, respectively (with *A*_pn_ = *A*_p_ + *A*_n_). 3D plots showing the relationships with b) maximum output power (*P*_max_), c) open‐circuit voltage (*V*_oc_), and d) internal resistance (*R*_in_). Variation of e) output power (*P*), and f) conversion efficiency (*η*) with current (*I*) at different Δ*T* for the 7 pairs of modules prepared. g) Comparison of experimental measurements and simulation results for *V*_oc_, *R*_in_, and *P*_max_ as a function of Δ*T*. The deviation from the simulated values increases with Δ*T*, likely due to the concomitant rise in *R*_in_. h) Continuous performance measurements of (Bi, Sb)_2_Te_3_‐based modules under a Δ*T* of ≈185 K. i) Comparison of the performance of *η*_max_ and *zT*_ave_ in this work with some reported data.^[^ ^8^, ^10^, ^11^, ^19^, ^20^, ^31^ ^]^

To ensure that high‐performance thermoelectric materials can be fully utilized in device applications, finite element analysis (FEA) was performed using the measured thermoelectric properties (see Figure S13, Supporting Information for the transport parameters of the *n*‐type material). The boundary conditions used in the simulations are detailed in the Supporting Information. As shown in **Figure** **6**a,b, the *η*_max_ and maximum output power (*P*_max_) were analyzed as functions of *H*/*A*_pn_ (with *A*_pn_ = *A*_p_ + *A*_n_, where *A*_p_ and *A*_n_ are the cross‐sectional areas of the *p*‐ and *n*‐type legs, respectively, and *H* is their leg height) and *A*_p_/*A*_n_. In general, *η*_max_ increased with *H*/*A*_pn_ due to the higher open‐circuit voltage (*V*_oc_) (Figure 6c), while *P*_max_ decreased gradually with *H*/*A*_pn_ due to an increase in internal resistance (*R*_in_), as shown in Figure 6d. At a fixed *H*/*A*_pn_, both *P*_max_ and *η*_max_ reached their peaks at specific *A*_p_/*A*_n_ ratios, ≈1.05 and 1.3, respectively, indicating that simultaneous optimization of *P*_max_ and *η*_max_ through *A*_p_/*A*_n_ adjustment alone is challenging. Notably, the dependence of *P*_max_ on *A*_p_/*A*_n_ was weaker than that of *η*_max_, as evidenced by the smoother, nearly parallel contours in Figure 6b, suggesting that *η*_max_ is more sensitive to variations in *A*_p_/*A*_n_. Based on these results, an *A*_p_/*A*_n_ ratio of 1.3 was selected to balance both *P*_max_ and *η*_max_. The *p*‐ and *n*‐type legs were designed with approximate cross‐sectional dimensions of 1.6 mm × 1.6 mm and 1.4 mm × 1.4 mm, respectively, yielding a leg height of 2.5 mm and an *H*/*A*_pn_ value of ≈0.55.

Based on the optimized device geometry, thermoelectric modules were successfully assembled. The linear voltage–current (*V*–*I*) characteristics shown in Figure S14a (Supporting Information) confirm stable device operation. The output power (*P*) of the module reached its maximum when the load resistance matched the *R*_in_. As illustrated in Figure 6e, *P*_max_ increased from 0.028 W at Δ*T* = 63 K to 0.19 W at Δ*T* = 182 K. At higher Δ*T*, parasitic losses such as those caused by contact resistance at the barrier layers resulted in a significant rise in the module's *R*_in_ relative to the simulated values, leading to a lower *P*_max_ (Figure 6f). In addition to contact resistance, other real‐world factors, including thermal interface degradation, packaging‐induced constraints, and lateral heat leakage may also contribute to the observed discrepancy, as these effects are not fully captured in the idealized simulation framework. Meanwhile, the heat flow (*Q*) measured at the cold side (Figure S14b, Supporting Information) exhibited a slight upward trend with increasing current, largely because the actual Δ*T* across the device was slightly smaller than the Δ*T* registered by the thermocouples, compounded by radiant heat losses from the heater. As a result, the experimentally measured device performance (Figure 6g, Δ*T* = 182 K) achieved a *η*_max_ of ≈6.9%, slightly lower than the simulated prediction. To confirm reliability, two additional devices (Figures S15, S16, Supporting Information) were evaluated, yielding highly reproducible results with a <5% error margin. Moreover, a 15 h continuous heating test at Δ*T* ≈ 185 K (Figure 6h) demonstrated negligible performance degradation. These results underscore that the efficiency of thermoelectric generation is governed by both the Carnot efficiency and the intrinsic thermoelectric properties of the materials:

(1) $$\eta_{\max}=\frac{T_{h}-T_{c}}{T_{h}}\;\frac{\sqrt{1+zT_{\mathrm{ave}}}-1}{\sqrt{1+zT_{\mathrm{ave}}}+\left(\frac{T_{c}}{T_{h}}\right)}$$

Using the relationship between *η*_max_ and *zT*_ave_, the power generation performance of the module and the comparative thermoelectric properties of the material are summarized in Figure 6i. The thermoelectric material achieved a *zT*_ave_ of ≈1.3 over the temperature range of 303–503 K, while the corresponding module reached a maximum *η*_max_ of ≈6.9% at Δ*T* = 182 K. Compared with other high‐performance systems, this work demonstrates a superior balance between material properties and device‐level performance.^[^ ^8^, ^10^, ^11^, ^19^, ^20^, ^31^ ^]^

## 3 Conclusion

In conclusion, we successfully enhanced the thermoelectric performance of *p*‐type Bi_2_Te_3_‐based alloys by simultaneously optimizing the *p*_H_ and microstructure through alloying with Cu_3_SbSe_3_. The homogenous dispersion of the dopant effectively tuned *p*_H_, while the reduction of micro‐holes and the formation of coherent TBs minimized *µ*_H_ loss, yielding a significant improvement in the *PF* across the entire temperature range, reaching ≈49 µW cm^−1^ K^−2^ at room temperature. Meanwhile, the introduction of scattering centers such as SFs and dislocations strengthened phonon scattering, leading to reduced *κ*. As a result, the optimized sample achieved a *zT*_max_ of ≈1.45 at 378 K, with an impressive *zT*_ave_ of ≈1.3 across 303–503 K. In addition, microstructural engineering substantially enhanced the mechanical properties, yielding a Vickers hardness of 96 Hv and a compressive strength of 187 MPa. A 7‐pair module fabricated from these optimized materials achieved a *η*_max_ of ≈6.9% at Δ*T* = 182 K. This work highlights the efficacy of combining microstructural engineering with targeted doping as a promising pathway for simultaneously optimizing the thermoelectric and mechanical performance of Bi_2_Te_3_‐based alloys, advancing their application in high‐performance thermoelectric devices.

## Conflict of Interest

The authors declare no conflict of interest.

## Supporting information



Supporting Information

## Data Availability

The data that support the findings of this study are available from the corresponding author upon reasonable request.
