# Supplementary material for: Cu3SbSe3‐Alloying‐Induced High Thermoelectric Performance and Mechanical Robustness in Bi2Te3‐Based Thermoelectric Materials
Source: Adv Sci (Weinh). 2025 Aug 19;12(42):e12417. doi: 10.1002/advs.202512417 (PMC12622560; doi:10.1002/advs.202512417)
Supplement: Supplementary file 1 — Supporting Information [file ADVS-12-e12417-s001.docx]

**Supplementary materials**

Cu_3_SbSe_3_-Alloying-Induced High Thermoelectric Performance and Mechanical Robustness in Bi_2_Te_3_-Based Thermoelectric Materials

*Ruiheng Li,* *Xiao-Lei Shi, Jianglong Zhu, Qian Deng, Wenxin Ou, Jie Zheng, Xiaobo Tan, Xuri Rao, Qiang Sun,* *Min Hong,* Ran Ang,* and Zhi-Gang Chen**

R. Li, J. Zhu, Q. Deng, W. Ou, J. Zheng, X. Tan, X. Rao, R. Ang

Key Laboratory of Radiation Physics and Technology, Ministry of Education, Institute of Nuclear Science and Technology, Sichuan University, Chengdu 610064, China

E-mail: rang@scu.edu.cn

X.-L. Shi, Z.-G. Chen

School of Chemistry and Physics, ARC Research Hub in Zero-emission Power Generation for Carbon Neutrality, and Centre for Materials Science, Queensland University of Technology, Brisbane, QLD, Australia.

E-mail: zhigang.chen@qut.edu.au

Q. Sun

State Key Laboratory of Oral Diseases, National Clinical Research Center for Oral Diseases, West China Hospital of Stomatology, Sichuan University, Chengdu, Sichuan 610041, China

M. Hong

Centre for Future Materials, University of Southern Queensland, Springfield Campus, QLD 4300, Australia

E-mail: min.hong@unisq.edu.au

R. Ang

Institute of New Energy and Low-Carbon Technology, Sichuan University, Chengdu 610065, China

College of Physics, Sichuan University, Chengdu 610064, China

Keywords: thermoelectric; bismuth telluride; alloying; mechanical property; conversion efficiency.

**Supplementary Methods**

1. **Sample synthesis**

High‑purity elemental Cu (wire, 5N, Aladdin), Sb (granules, 5N, Aladdin), and Se (granules, 5N, Aladdin) were weighed according to the stoichiometric composition of Cu_3_SbSe_3_. The raw materials were sealed in quartz ampoules under a vacuum of ~10^–3^ Torr, heated to 1273 K for 24 h, and then quenched in ice water. The resulting ingots were annealed at 623 K for 5 days. The phase structure of the synthesized Cu_3_SbSe_3_ was examined by X‑ray diffraction (XRD), as shown in **Figure S1**, and the main diffraction peaks were well‑indexed to the standard PDF card (PDF#97‑040‑1095).

To prepare the thermoelectric materials, pre‑synthesized Cu_3_SbSe_3_, Bi (granules, 4N, Aladdin), Sb (granules, 5N, Aladdin), and Te (chunks, 5N, Aladdin) were weighed in stoichiometric proportions and thoroughly mixed. The mixture was sealed in a quartz ampoule, flame‑sealed, and heated to 1073 K over 5 h, held at this temperature for 6 h, and then furnace‑cooled to room temperature over 10 h. The resulting ingot was transferred to a stainless‑steel jar filled with argon gas and ball‑milled for 30 minutes at a vibration frequency of 1800 r/min. The powder was then placed into a Ø12.7 mm graphite die and hot‑pressed at 723 K for 15 minutes under vacuum (<5 Pa) and a uniaxial pressure of 50 MPa. The final compact achieved a relative density exceeding 95% of the theoretical value.

1. **Structural and mechanical properties characterization**

The phase purity and crystal structure of the samples were examined by X‑ray diffraction (XRD) using Cu Kα radiation. Morphological and compositional analyses were conducted using a scanning electron microscope (SEM, FEI Inspect F50) equipped with energy‑dispersive X‑ray spectroscopy (EDS) for detailed elemental mapping. Transmission electron microscopy (TEM, Philips Tecnai F20, equipped with energy-dispersive spectroscopy EDS for compositional analysis) was used to characterize the microstructure. The microhardness (Hv) was measured with a Vickers diamond indenter on an HVS‑1000 tester, applying a load of 2 N for 10 s to ensure accurate results. The compressive stress–strain behavior of the bulk samples was evaluated using a universal testing machine at a loading rate of 0.5 mm/min.

1. **Thermoelectric property measurements：**

The electrical transport properties were measured using a CTA‑Pro system (Beijing Cryoall Science and Technology Co., Ltd.). Thermal diffusivity was determined *via* the laser flash method with a NETZSCH LFA‑467 HT apparatus. The thermal conductivity (*κ*) was calculated using the relation *κ* = *D* × *C*_p_ × *ρ*, where *C*_p_ (specific heat) was estimated based on the Dulong–Petit law and *ρ* is the geometrical density of the sample. The Hall mobility (*μ*_H_) and carrier concentration (*p*_H_) were measured using the van der Pauw method under a reversible magnetic field of 1.5 T. The electronic thermal conductivity (*κ*_e_) was estimated via the Wiedemann–Franz law, *κ*_e_ = *LσT*, with the Lorenz number (*L*) calculated from *L* = [1.5 + exp(-|*S*|/116)] × 10^–8^ V^2^ K^–2^.^[1]^ All electrical and thermal transport properties were measured along the direction parallel to the hot‑pressing (HP) pressure. The uncertainties for electrical conductivity (*σ*) and Seebeck coefficient (*S*) were ±2% and ±5%, respectively, while the uncertainty for *κ* was approximately ±3%, yielding an overall error of roughly 10% in the *zT* value.

1. **Calculation methods**

Density functional theory (DFT) calculations were performed using the Vienna Ab initio Simulation Package (VASP),^[2-4]^ with post-processing analysis conducted via the VASPKIT package.^[5]^ The electron exchange-correlation was described by the Perdew, Burke, and Ernzerhof (PBE) functional within the generalized gradient approximation (GGA).^[6]^ Starting with the rhombohedral primitive cell of Sb_2_Te_3_, a 3 × 3 × 3 supercell was constructed. To simulate the (Bi, Sb)_2_Te_3_ alloy in this study, 10 Sb atoms were randomly substituted with Bi atoms, resulting in a composition of Bi_10_Sb_44_Te_81_. To simulate Cu and Se doping, one Sb atom was randomly replaced by Cu, and one Te atom was replaced by Se, producing the structure Bi_10_Sb_43_CuTe_80_Se. The structures were fully relaxed until the total energy converged to < 10^-5^ eV and the force on each atom was < 0.01 eV Å^-1^. The calculated band structures of the supercells were unfolded to the primitive Brillouin zone following the high-symmetry path.

1. **Construction of TE module and evaluation of power generation performance**

A full‑scale thermoelectric module was fabricated using p‑type Bi_0.4_Sb_1.6_Te_3.01_ + 0.04 wt.% Cu_3_SbSe_3_, prepared *via* high‑energy ball milling (HBM) and HP, and n‑type Bi_2_Te_2.7_Se_0.3_, synthesized by hot extrusion. The module comprised 7 p–n leg pairs, each measuring 10 mm × 10 mm. The module geometry was optimized using the commercial finite element analysis (FEA) software COMSOL Multiphysics. The p‑ and n‑type legs were designed with dimensions of 1.6 mm × 1.6 mm × 2.5 mm and 1.4 mm × 1.4 mm × 2.5 mm, respectively. Both leg types were electroplated with a nickel (Ni) diffusion barrier and alternately arranged on a Cu‑clad Al_2_O_3_ substrate. Sn_90_Sb_10_ solder (melting point ~260 °C) was used as the bonding material. The power generation performance of the module was evaluated using a custom‑built testing system, calibrated and validated against a Mini‑PEM system from Advance Riko, Japan.

1. **The effective mass modeling**

By treating the band structure as a single parabolic band and considering acoustic phonon scattering.^[7]^ The *S* can be expressed as:

$S=\frac{k_{B}}{e}[\frac{\left( r+5/2 \right)F_{r+3/2}\left( \eta\right)}{\left( r+3/2 \right)F_{r+1/2}\left( \eta\right)}-\eta]$ (S1)

where *η* is the reduced chemical potential, *k*_B_ is the Boltzmann constant, *e* is the electron charge, and *r* is the scattering factor (with *r* = -1/2).

$n_{H}=\frac{16\pi\left( 2m_{d}^{*}k_{B}T \right)^{3/2}}{3h^{3}}\frac{F_{0}^{2}}{F_{-1/2}}$ (S2)

$\mu_{H}=\mu_{0}\frac{1}{2}\frac{F_{-1/2}}{F_{0}}$ (S3)

Here the density of states effective mass $m_{d}^{*}$, which is obtained from the Pisarenco plot. The electrical conductivity (*σ*) can then be written as:

$\sigma=\frac{8\pi e\left( 2k_{B}T \right)^{3/2}m_{d}^{*3/2}\mu_{0}}{3h^{3}}F_{0}=\frac{8\pi e\left( 2k_{B}T \right)^{3/2}m_{e}^{-3/2}\mu_{w}}{3h^{3}}F_{0}$ (S4)

where $\mu_{w}=\left( m_{d}^{*}/m_{e} \right)^{3/2}\mu_{0}$. The Lorentz factor (*L*) is given by

$L=(\frac{k_{B}}{e})^{2}\frac{3F_{0}F_{2}-4F_{1}^{2}}{F_{0}^{2}}$ (S5)

In these equations, the integral $F_{j}$ is defined by:

$F_{j}\left( \eta\right)=\int_{0}^{\infty} \frac{\xi^{j}d\xi}{1+e^{(\xi-\eta)}}$ (S6)

1. **The weighted mobility *μ*_w_**

The weighted mobility *μ*_w_ primarily reflects the electrical transport properties and is calculated using the following equation:^[8]^

$\mu_{w}=\frac{3h^{3}\sigma}{8\pi e(2m_{e}k_{B}T)^{3/2}}[\frac{exp[\frac{|S|}{k_{B}/e}-2]}{1+exp[-5(\frac{|s|}{k_{B}/e}-1)]}+\frac{\frac{3|S|}{\pi^{2}k_{B}/e}}{1+exp[5(\frac{|S|}{k_{B}/e}-1)]}]$ (S7)

1. **The calculation of lattice thermal conductivity (*κ*_l_)**

The integrand term, along with the coefficient in Equation (S8), represents the spectral lattice thermal conductivity:^[9]^

$\text{κ}_{s}(\omega)=\frac{k_{B}}{2\pi^{2}\upsilon}\left( \frac{k_{B}T}{\hbar} \right)^{3}\tau_{tot}(x)\frac{x^{4}e^{x}}{{(e^{x}-1)}^{2}}$ (S8)

In the above equation, *x* = *ћω/k_B_T* is the reduced phonon frequency, $k_{B}$ is the Boltzmann constant, $\upsilon$ is the average sound velocity, calculated using $\upsilon={[\frac{1}{3}\left( \frac{1}{\upsilon_{L}^{3}}+\frac{1}{\upsilon_{T}^{3}} \right)]}^{\frac{-1}{3}}$, where $\upsilon_{L}$ and $\upsilon_{T}$ are the longitudinal and transverse sound velocities, respectively. $\hbar$ is reduced Plank’s constant, $\theta_{D}$ is the Debye temperature, and $\omega$ is the phonon frequency. The total relaxation time $\tau_{tot}$is calculated according to Matthiessen’s rule:^[10]^

$\tau_{tot}^{-1}=\tau_{UN}^{-1}+\tau_{PD}^{-1}+\tau_{GB}^{-1}+\tau_{SF}^{-1}$ (S9)

where *τ_UN_*, *τ_PD_*, *τ_GB,_* and *τ_SF_* represent the relaxation times of the Umklapp process, point defect scattering, grain boundary scattering, and stacking faults, respectively. The relevant phonon relaxation times are given by:

Umklapp phonon-phonon scattering:

$\tau_{UN}^{-1}=A_{N}\frac{2}{\left( 6\pi^{2} \right)^{\frac{1}{3}}}\frac{k_{B}\bar{V}^{\frac{1}{3}}\gamma^{2}\omega^{2}T}{\bar{M}\upsilon^{3}}$ (S10)

Point defect phonon scattering:

$\tau_{PD}^{-1}=\frac{\bar{V}\omega^{4}}{4\pi\upsilon^{3}}\Gamma$ (S11)

Grain boundary phonon scattering:

$\tau_{GB}^{-1}=\frac{\upsilon}{d}$ (S12)

In these equations, *γ* is the Grüneisen parameter, $\bar{M}$ is the average mass, *m** is the effective mass of the charger carrier, *ρ* is the sample density, $\bar{V}$ is the average atomic volume, *Γ* is the point defect scattering parameter (which is determined by considering only mass difference, a method known to work well for the Bi_2_Te_3_-Sb_2_Te_3_ system), and *d* is the grain size. The Umklapp phonon–phonon scattering strength coefficient *A*_N_ was fitted to experimental data of the in-plane *κ_l_*.

Considering only the specular reflection of phonons at stacking faults, Klemens found that:^[11]^

$\tau_{SF}^{-1}=0.7\frac{a^{2}}{v}\gamma^{2}\omega^{2}N_{s}$ (S13)

where a, $v$, $\gamma$ and $N_{s}$ are lattice parameter, average sound speed, Grüneisen parameter, and the number of stacking faults crossing a unit length, respectively.

Dislocation scattering includes both dislocation core (*τ*_DC_) and dislocation strain (*τ*_DS_) scattering:

${}_{D}^{-1}=\tau_{DC}^{-1}+\tau_{DS}^{-1}$ (S14)

${}_{DC}^{-1}=N_{D}\frac{\bar{V}^{4/3}}{\upsilon^{2}}{}^{3}$ (S15)

${}_{DS}^{-1}=0.6N_{D}{}^{2}{B_{D}}^{2}\left\{ \frac{1}{2}+\frac{1}{24}\left( \frac{1-2}{1-} \right)^{2}\left[ 1+\sqrt{2}\left( \frac{\upsilon_{L}}{\upsilon_{T}} \right)^{2} \right]^{2} \right\}$ (S16)

In the above equations, *N_D_* is the number of dislocations crossing a unit length and *B*_D_ is the magnitude of the Burgers vector of the dislocation.

The detailed parameters are listed in **Table S2**.

Sound velocity testing is conducted using a sound velocity measuring instrument. An ultrasonic signal generator emits different types of ultrasonic waves, which are reflected off the back of the sample. The reflected sound waves then pass through the sample and are received by the instrument. By measuring the distance (*d*) between the two ends of the sample and the total time (*t*) taken for the ultrasonic waves to travel back and forth through the material, the sound velocity *ν* can be calculated as *ν* = 2*d*/*t*.The average sound velocity ($\nu$) can be derived from the longitudinal ($\nu_{L}$) and transverse ($\nu_{T}$) sound velocities by the relationship: ^[12]^

$\nu={[\frac{1}{3}(\frac{1}{\nu_{L}^{3}}+\frac{2}{\nu_{T}^{3}})]}^{-1/3}$ (S17)

The Grüneisen parameter *γ* can be derived from the Poisson ratio ($r$) using the following equation:^[13]^

$\gamma=\frac{3}{2}\left( \frac{1+r}{2-3r} \right)$ (S18)

where the Poisson ratio *υ_p_* can be calculated from the longitudinal (*v_L_*) and transverse (*v_T_*) sound velocities by the following relationship: ^[13]^

$r=\frac{1-2\left( {v_{T}}/{v_{L}} \right)^{2}}{2-2\left( {v_{T}}/{v_{L}} \right)^{2}}$ (S19)

1. **3D finite element model**

The main constitutive equations associated with the TE conversion in TE coupling are as follows:

$J=-\sigma\alpha\nabla T-\sigma\nabla V$ (S20)

$q=T\alpha J-J\nabla V-\kappa\nabla T$ (S21)

Equation (S20) accounts for the contribution of the Seebeck effect to current density. Equation (S21) describes the combined effects of the Seebeck effect, Peltier effect, and Joule heating on heat flux.

Under steady-state conditions, the divergence of the current density is zero, and the corresponding boundary conditions can be formulated as:

$\nabla\cdot J=0$ (S22)

$\nabla\cdot q=-J\nabla V$ (S23)

Here, $J\nabla V$ represents the Joule heat flux generated by the circuit.

More explicitly, the TE equations are:

$\nabla\cdot(-\sigma\alpha\nabla T-\sigma\nabla V)=0$ (S24)

$\nabla\cdot(\alpha J-J\nabla V-\kappa\nabla T)=-(-\sigma\nabla V-\sigma\alpha\nabla T)\cdot(\nabla V)$ (S25)

By specifying a given current value, the potential and temperature distributions can be obtained by solving the equations above. The output voltage (*V*), output power (*P*), and heat absorption on the hot side (*Q*_h_) were calculated using mathematical operations. Thermoelectric conversion efficiency (*η*) was determined using the following equation:

$\eta=\frac{P}{Q_{h}}=\frac{IV}{Q_{h}}\times100\%$ (S26)

The *I*-*P* and *I*-*η* curves can be obtained by varying the current in the system, and *P*_max_ and *η*_max_ are achieved by optimizing the current.

**Supplementary Figures**


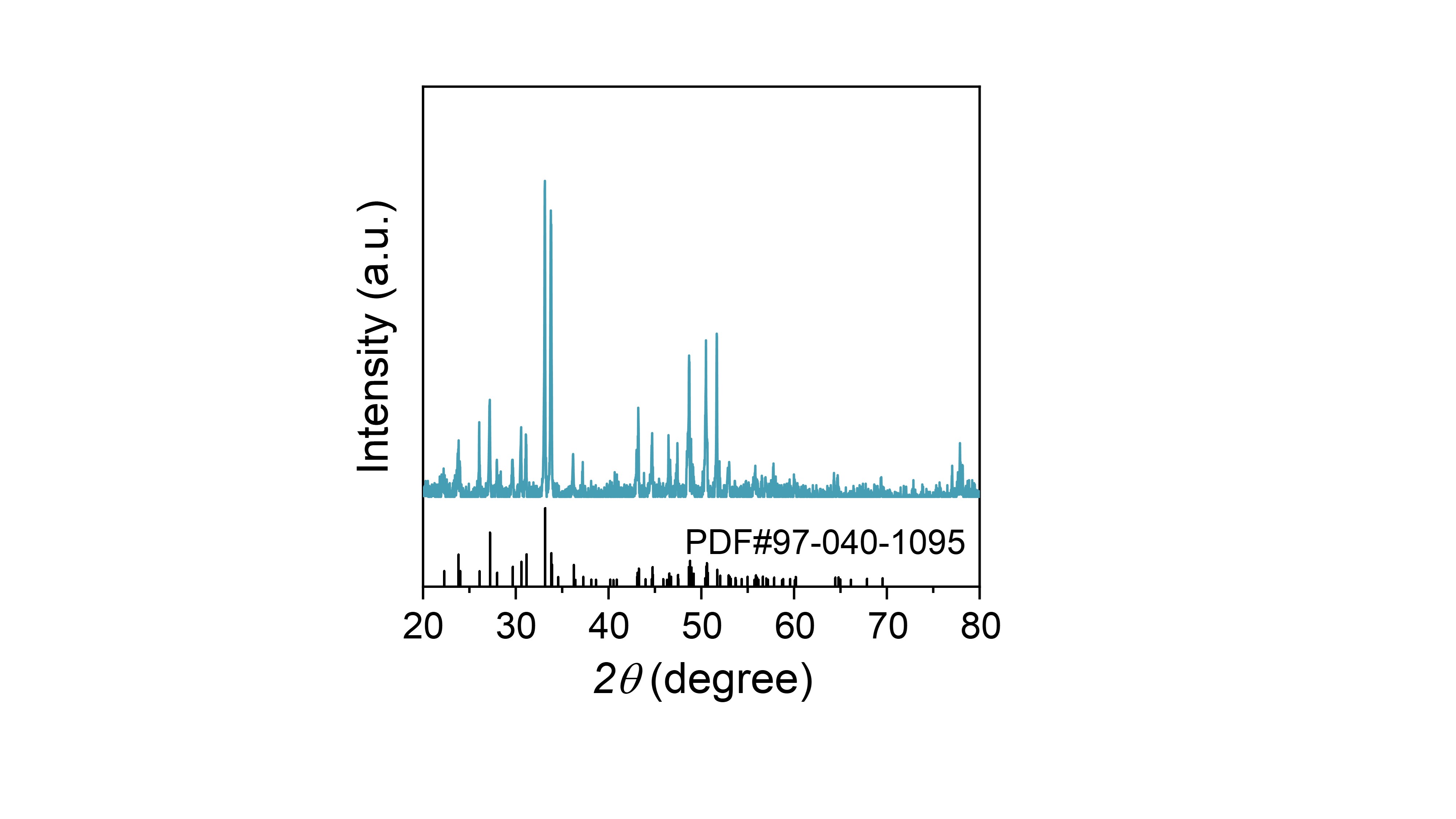


**Figure S1** X‑ray diffraction (XRD) pattern of Cu_3_SbSe_3_ sample.


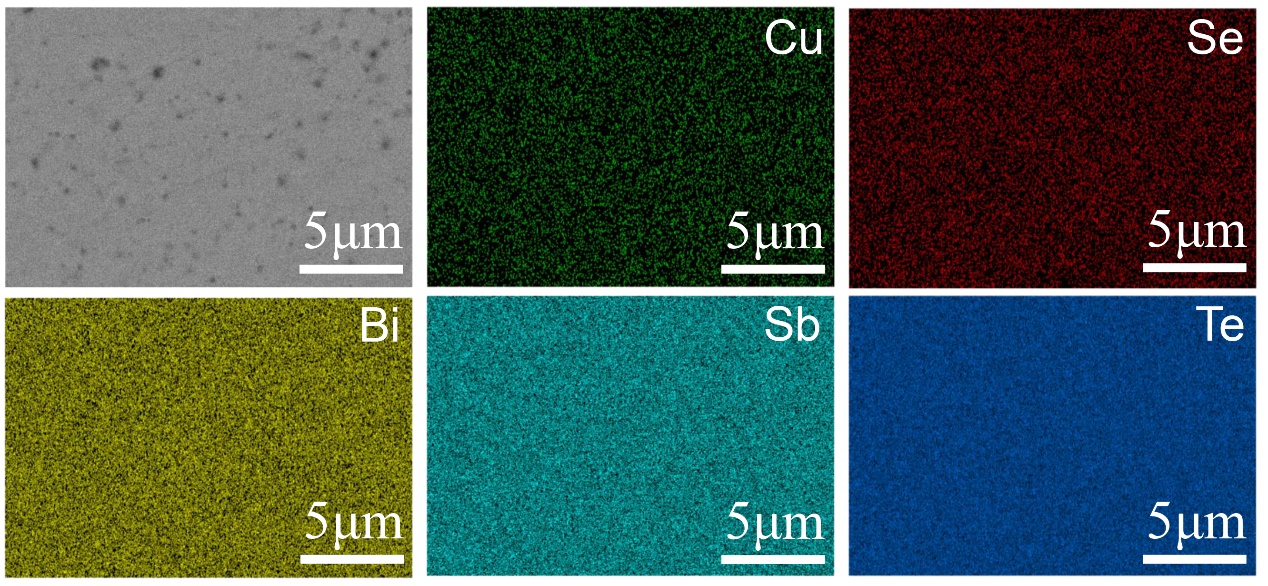


**Figure S2** Scanning electron microscope (SEM) image and corresponding energy dispersive spectroscopy (EDS) spectrum of the Bi_0.4_Sb_1.6_Te_3.01_ + 0.04 wt. % Cu_3_SbSe_3_.


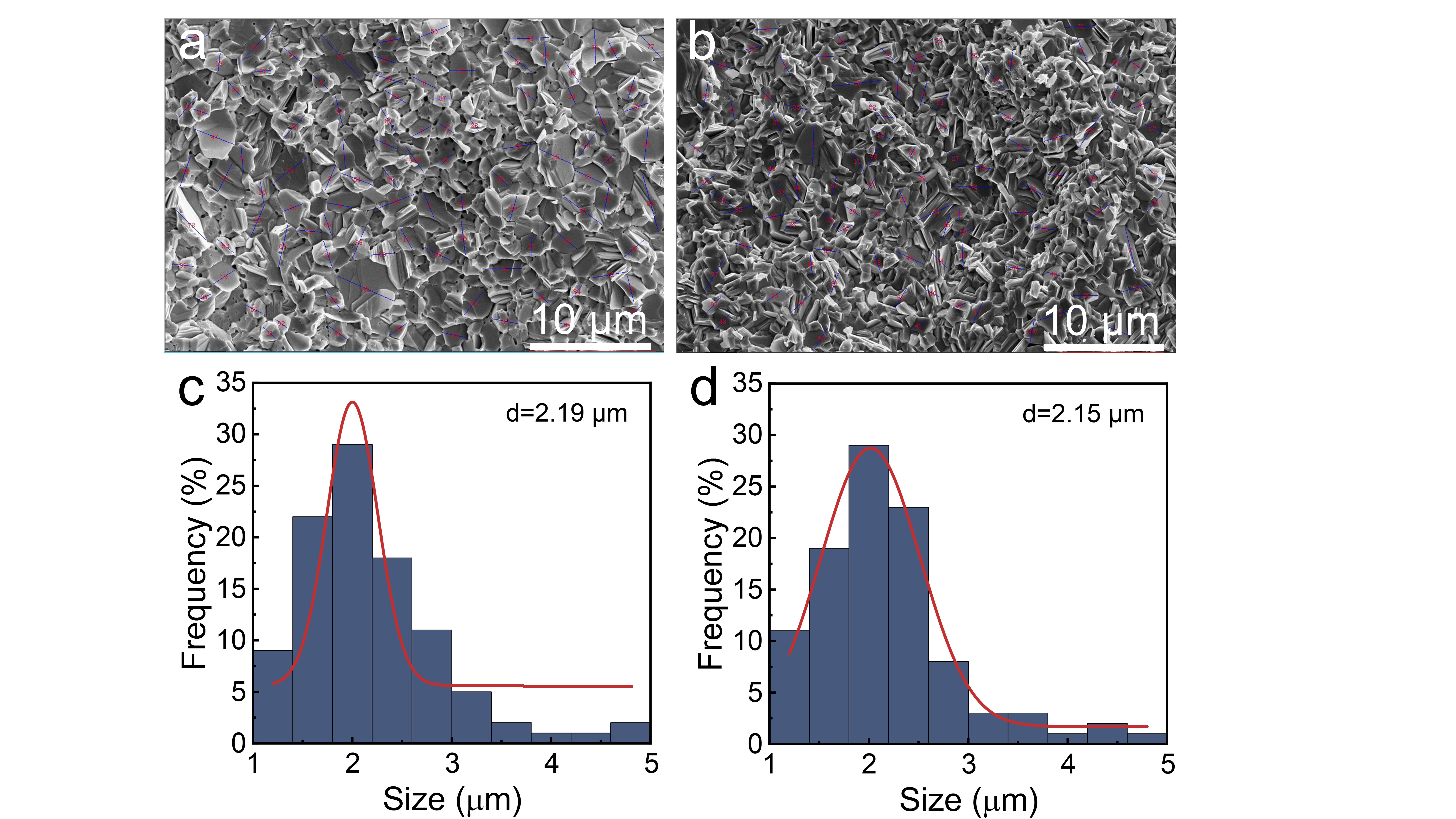


**Figure S3** Fracture surface images of (a) Bi_0.4_Sb_1.6_Te_3.01_ and (b) Bi_0.4_Sb_1.6_Te_3.01_ + 0.04 wt.% Cu_3_SbSe_3_; Corresponding grain size distributions of (c) Bi_0.4_Sb_1.6_Te_3.01_ and (d) Bi_0.4_Sb_1.6_Te_3.01_ + 0.04 wt.% Cu_3_SbSe_3_.


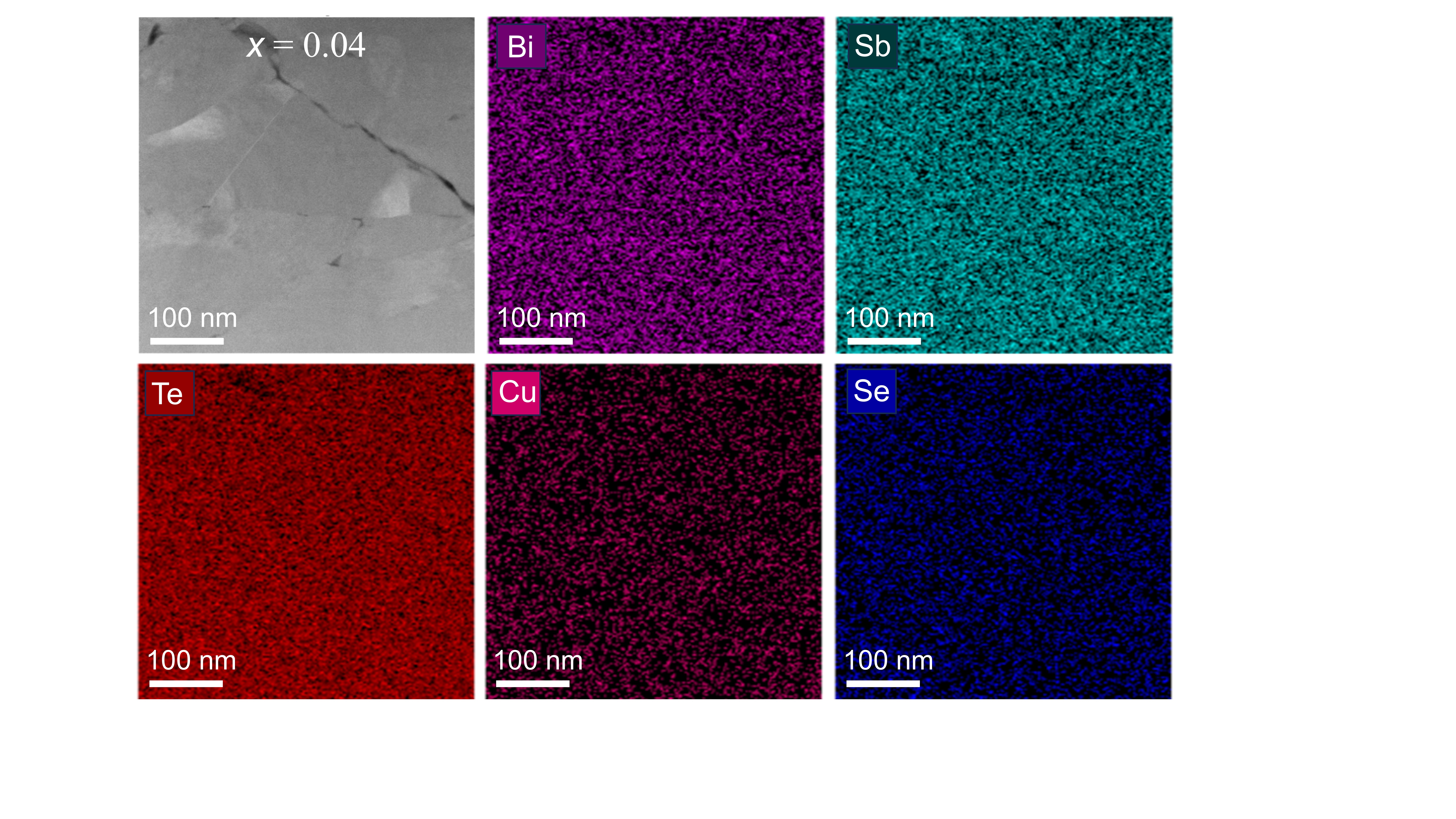


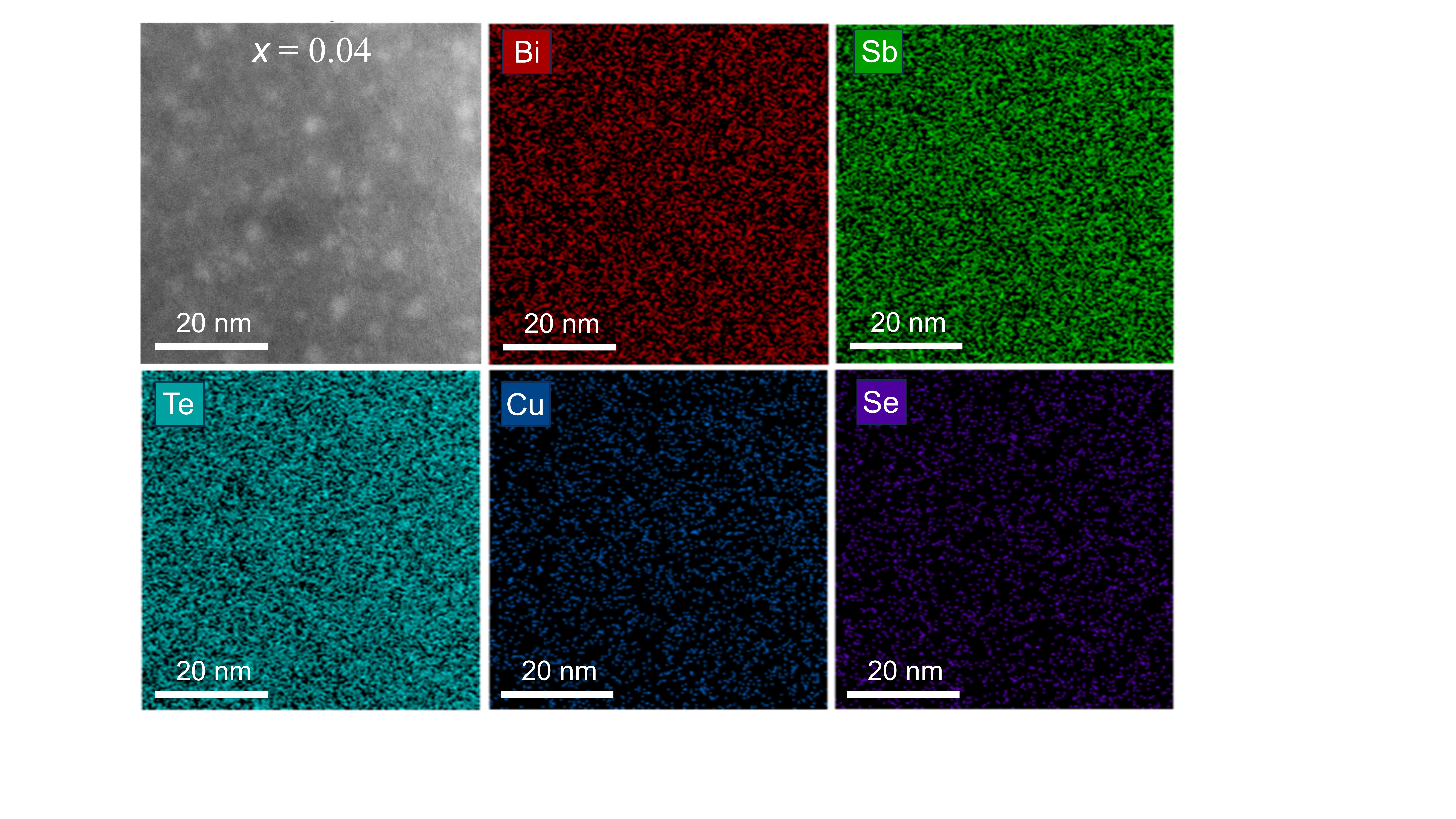


**Figure S4** Low-magnification scanning transmission electron microscopy (STEM) image and corresponding EDS mappings of the Bi_0.4_Sb_1.6_Te_3.01_ + 0.04 wt.% Cu_3_SbSe_3_.


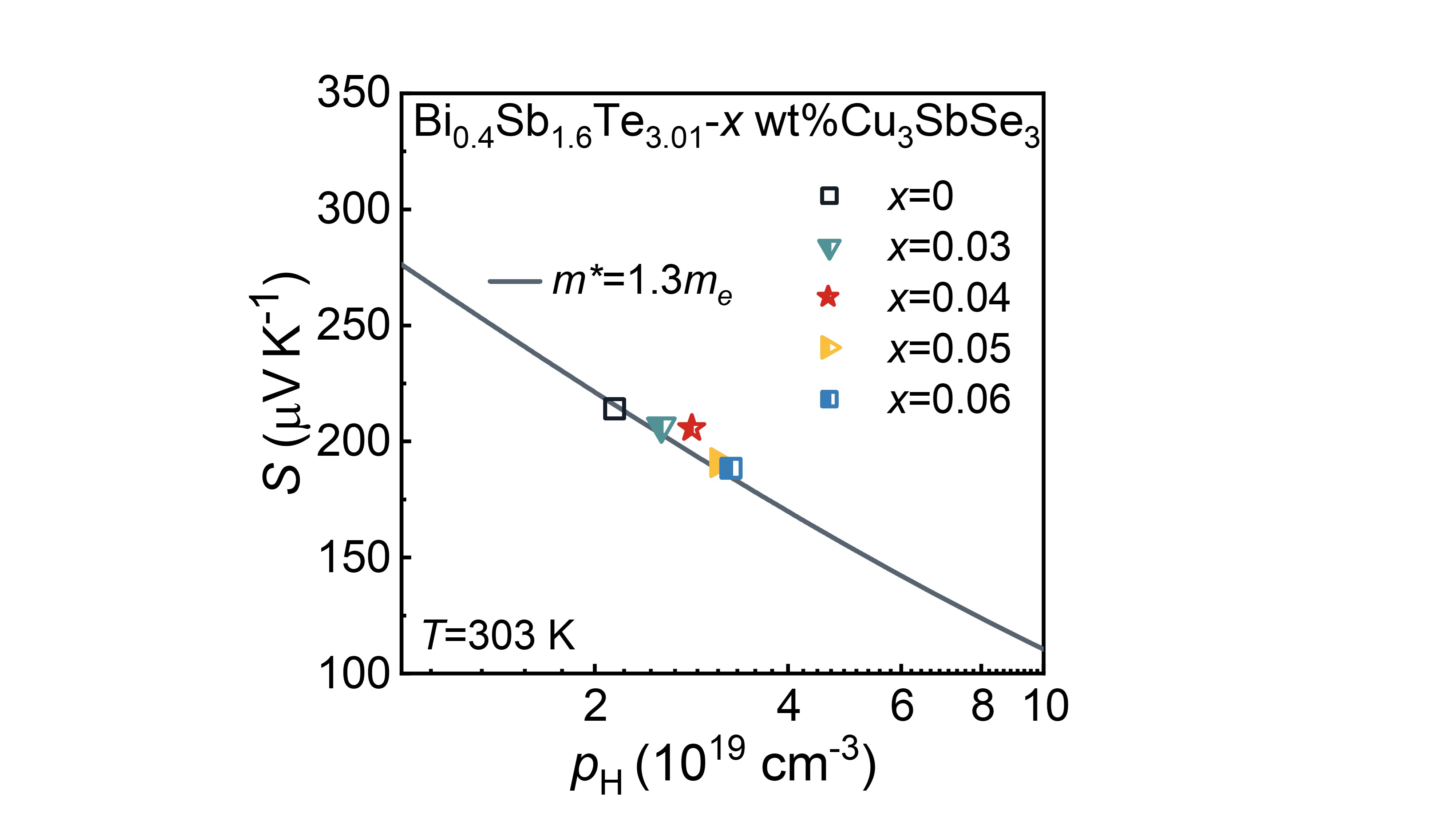


**Figure S5** Pisarenko curve at 303 K for the Bi_0.4_Sb_1.6_Te_3.01_ + *x* wt.%Cu_3_SbSe_3_ sample, using the effective mass model.


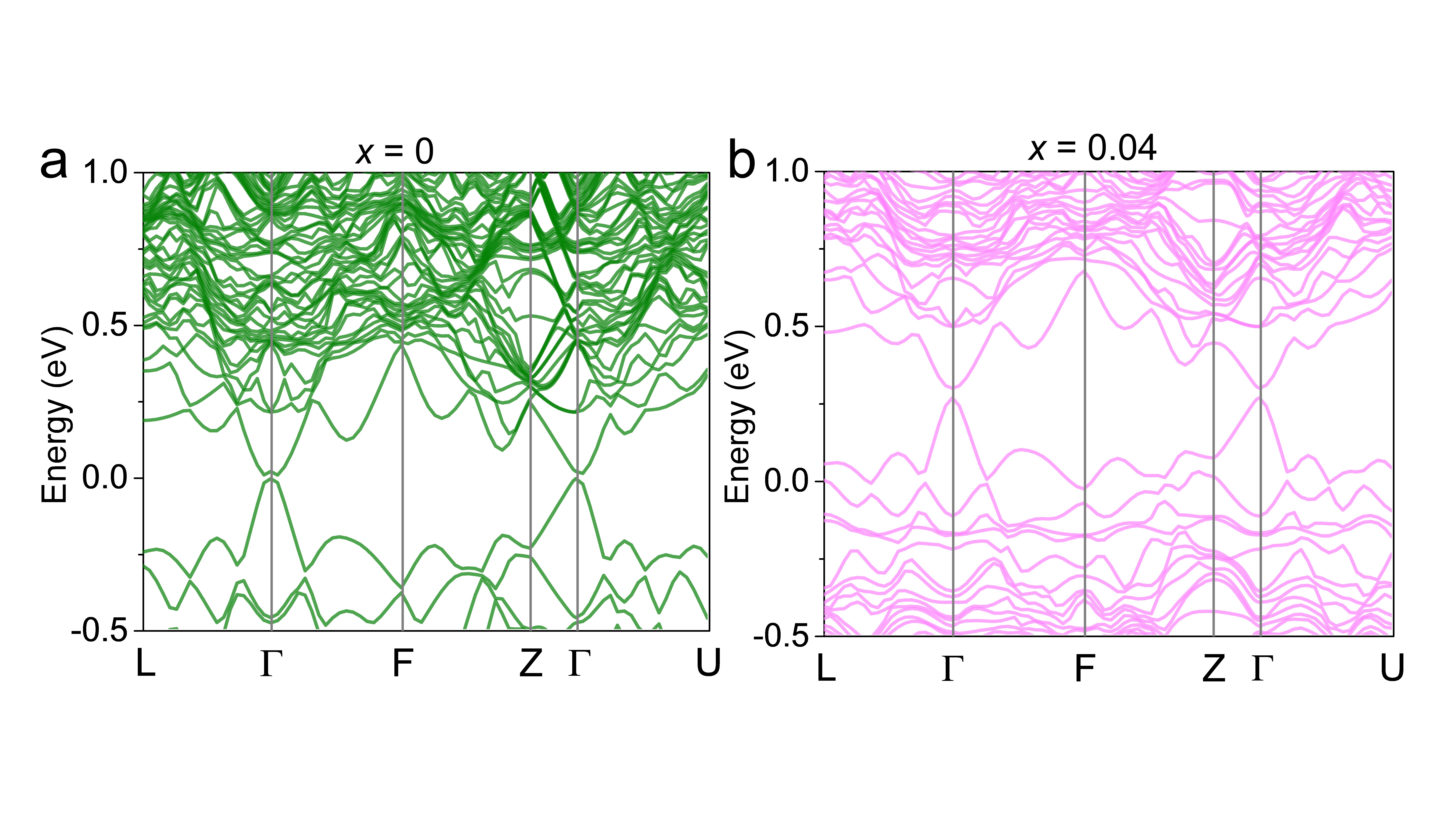


**Figure S6** Band structures calculated for (a) Bi_0.4_Sb_1.6_Te_3.01_ and (b) Bi_0.4_Sb_1.6_Te_3.01_ + 0.04 wt.% Cu_3_SbSe_3_.


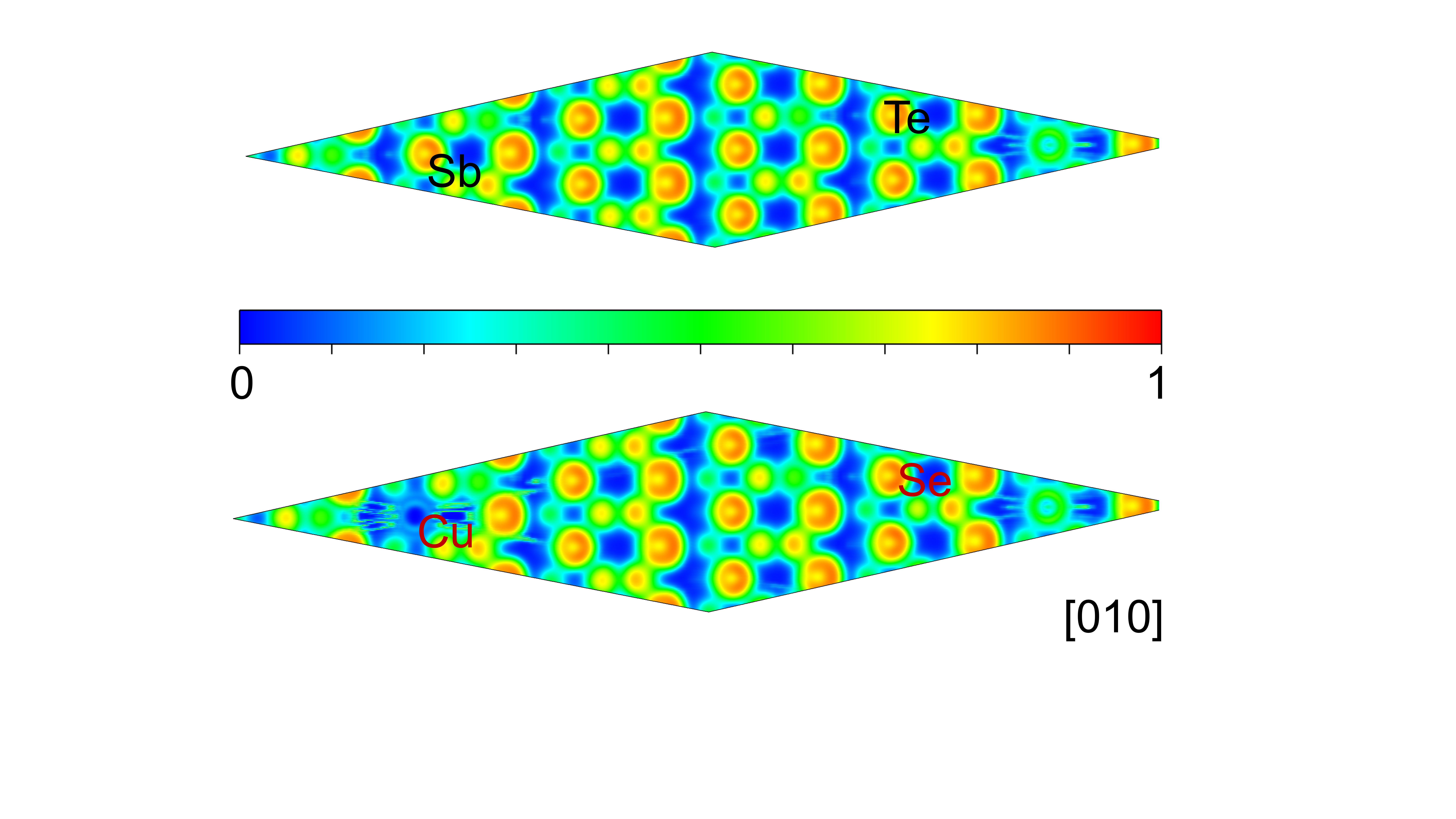


**Figure S7** 2D mappings of the calculated electron localization functions (ELF) for Bi_0.4_Sb_1.6_Te_3.01_ and Bi_0.4_Sb_1.6_Te_3.01_ +0.04 wt.% Cu_3_SbSe_3_.





**Figure S8** Structural models of (a) Bi_0.4_Sb_1.6_Te_3.01_ and (b) Bi_0.4_Sb_1.6_Te_3.01_ +0.04 wt.% Cu_3_SbSe_3_ for the ELF and DFT calculations.


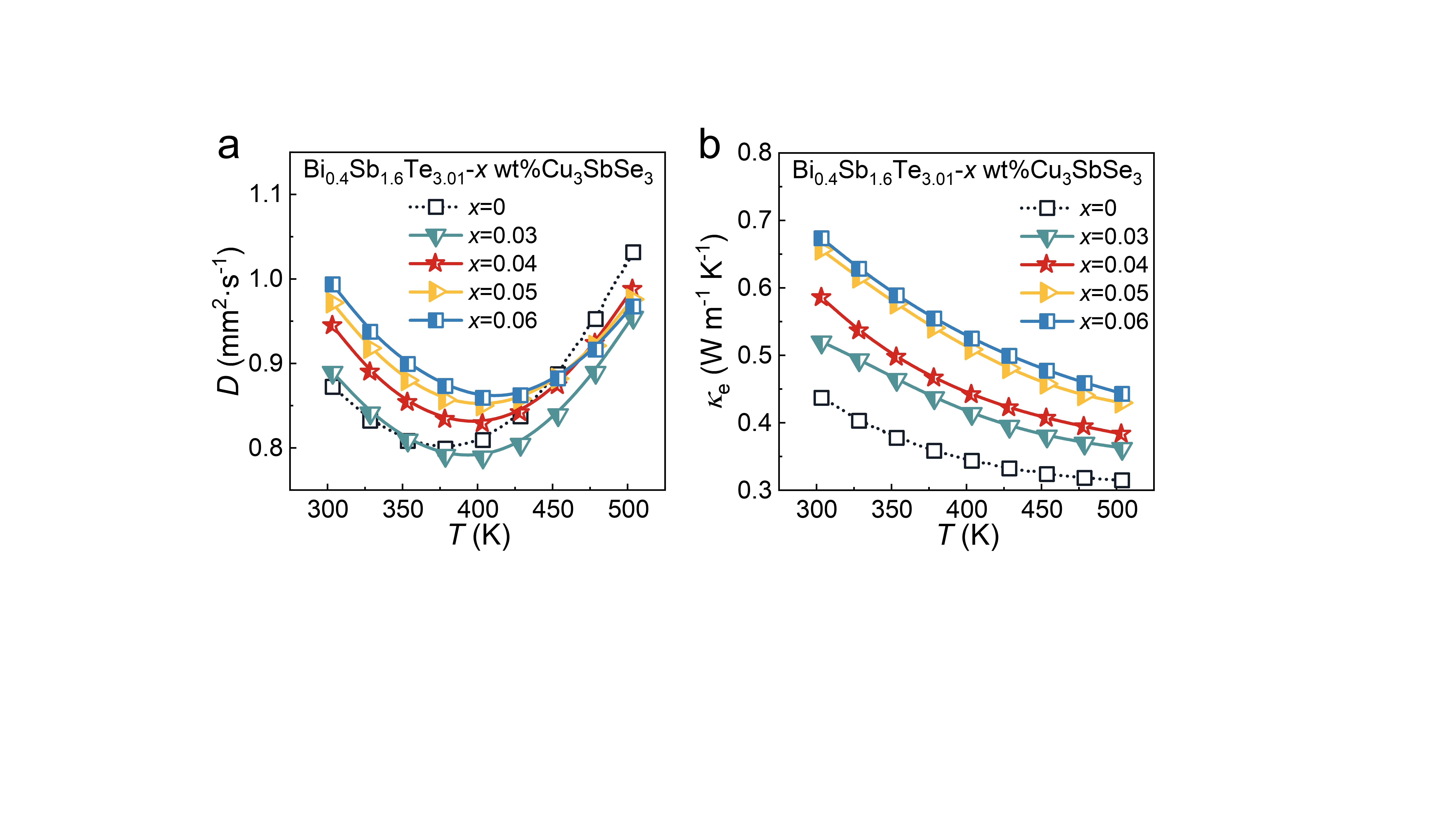


**Figure S9** (a) thermal diffusivity (*D*) and (b) electronic thermal conductivity (*κ*_e_) of Bi_0.4_Sb_1.6_Te_3.01_ + *x* wt.%Cu_3_SbSe_3_.


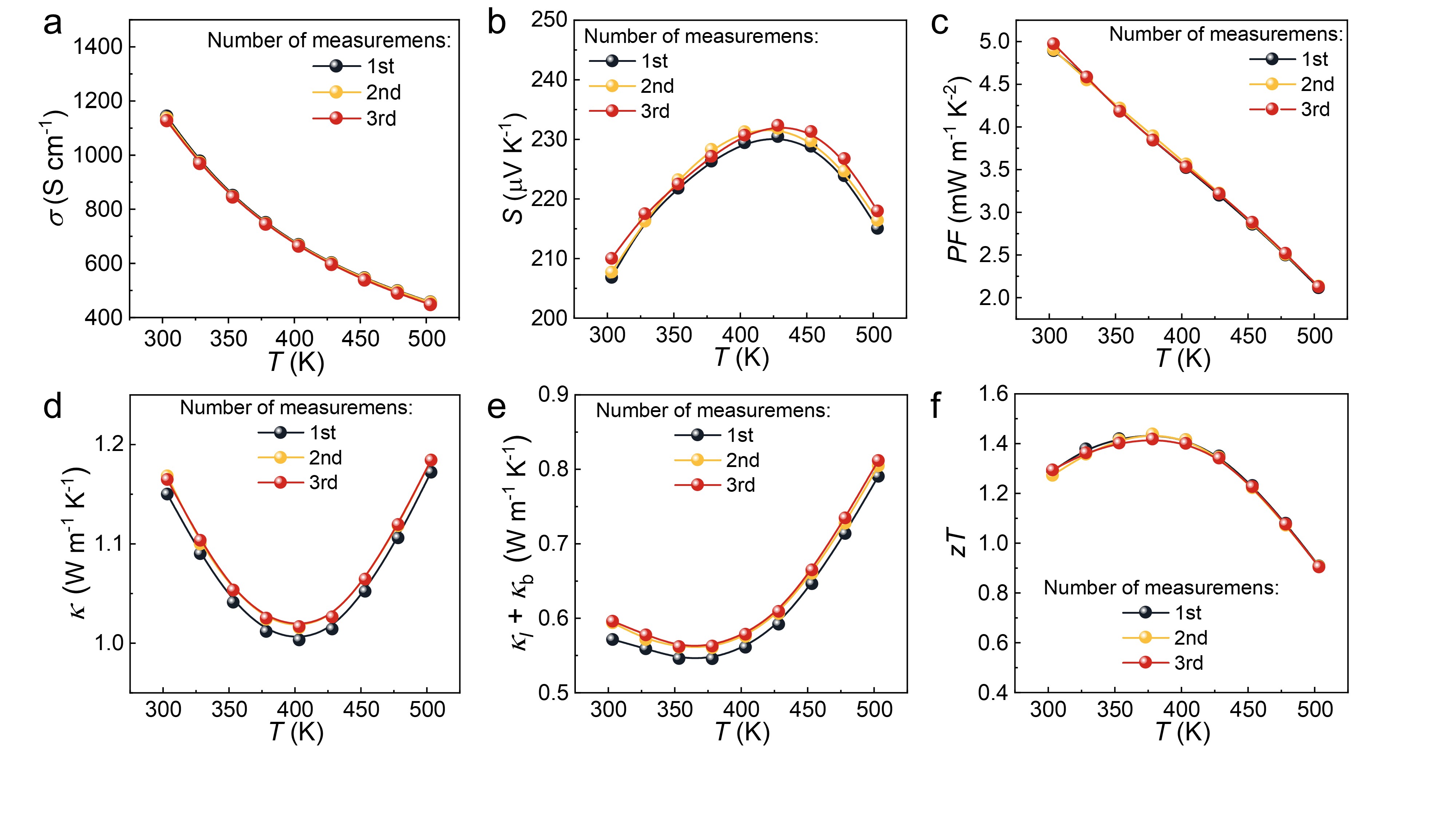


**Figure S10** Data from repeated measurements for Bi_0.4_Sb_1.6_Te_3.01_ + 0.04 wt.% Cu_3_SbSe_3_ sample. Temperature dependence of (a) electrical conductivity (*σ*), (b) Seebeck coefficient (*S*), (c) power factor (*PF*), (d) *κ*, (e) *κ*_l_+*κ*_b_, and (f) *zT*.


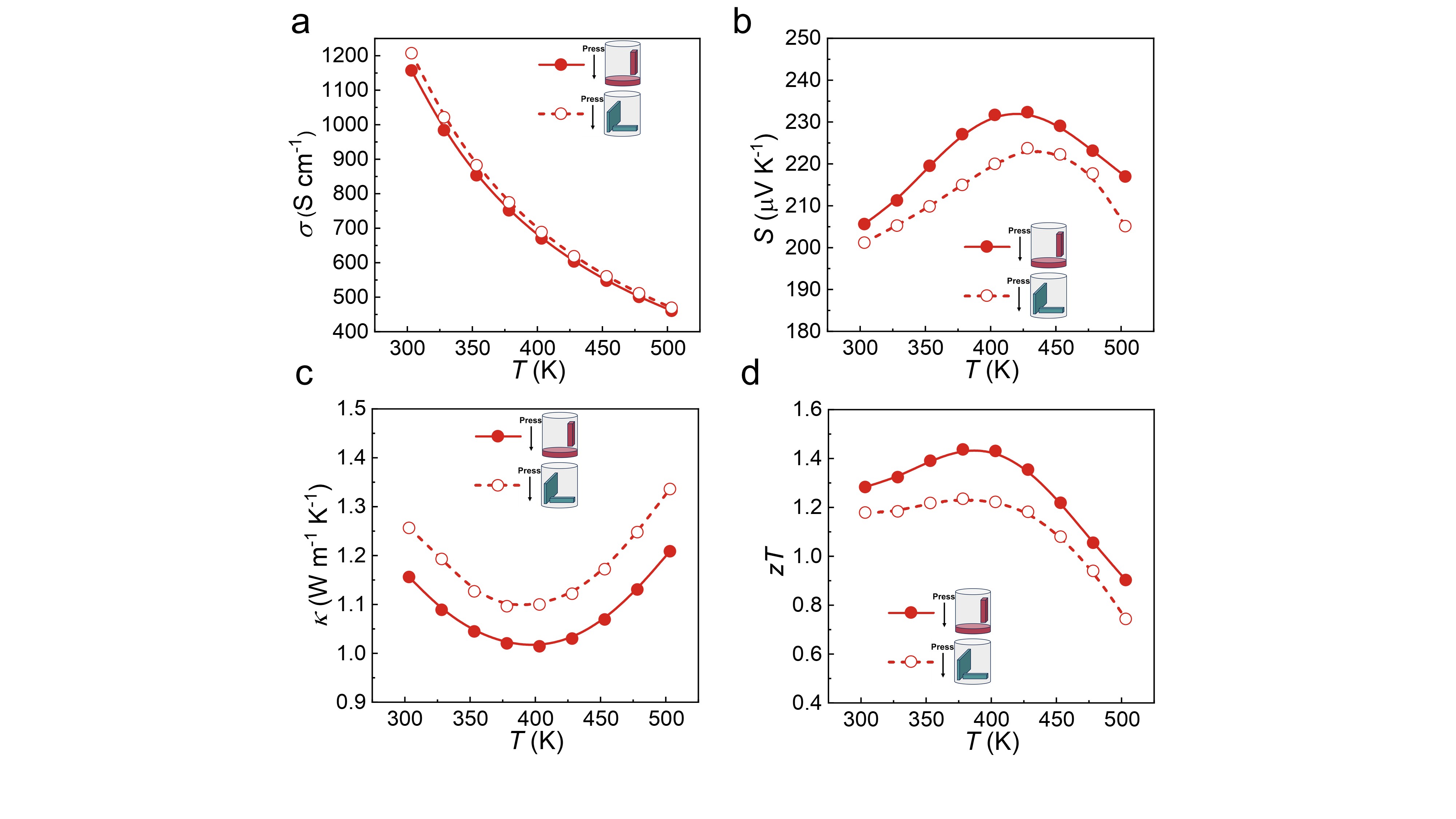


**Figure S11** Comparison of the thermoelectric properties of Bi_0.4_Sb_1.6_Te_3.01_ + 0.04 wt.% Cu_3_SbSe_3_ samples measured parallel and perpendicular to the hot-pressing direction: (a) *σ*, (b) *S*, (c) *κ*, and (d) *zT*.


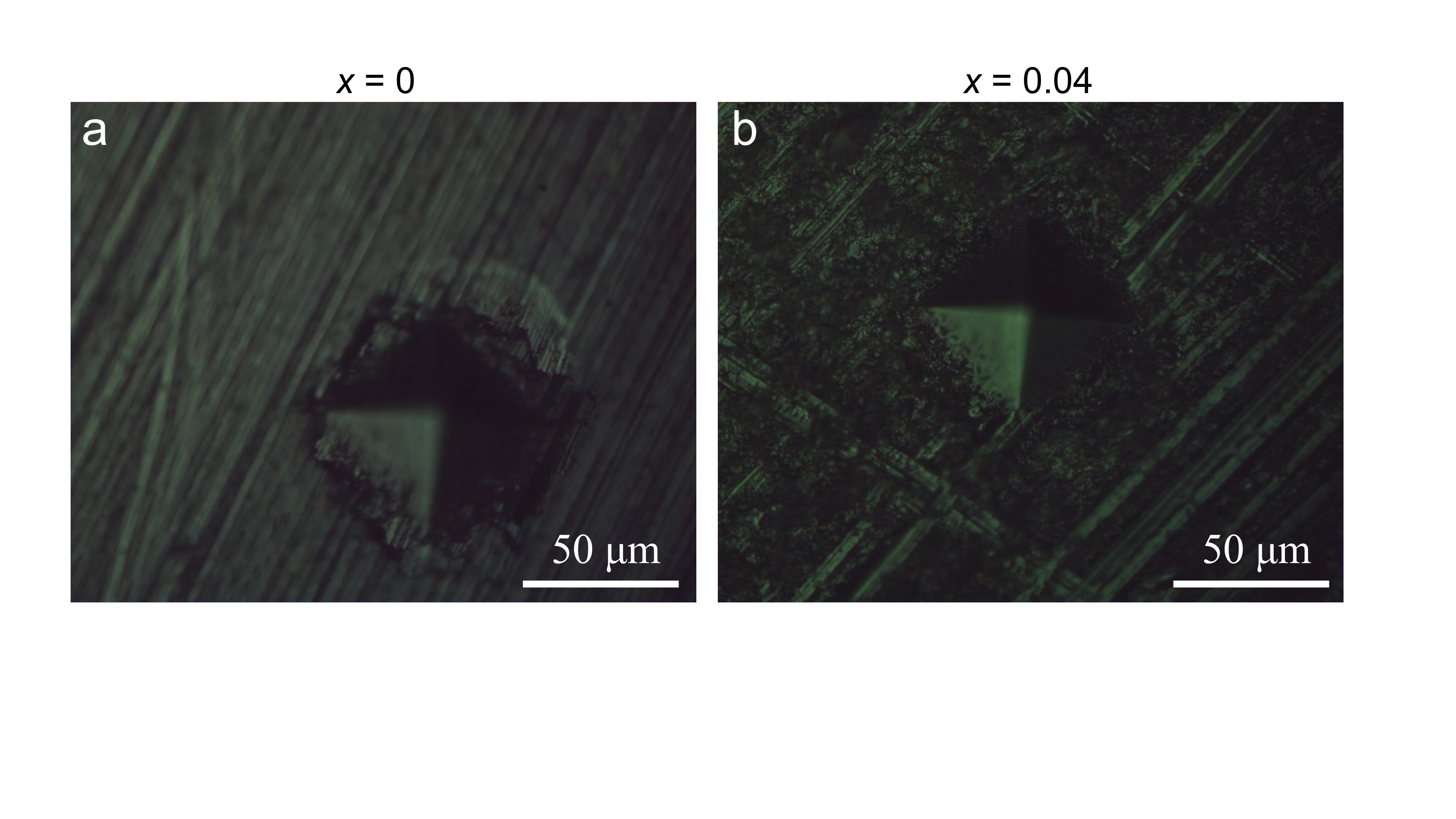


**Figure S12** Indentation images of Vickers microhardness tests for (a) *x* = 0 and (b) *x* = 0.04.


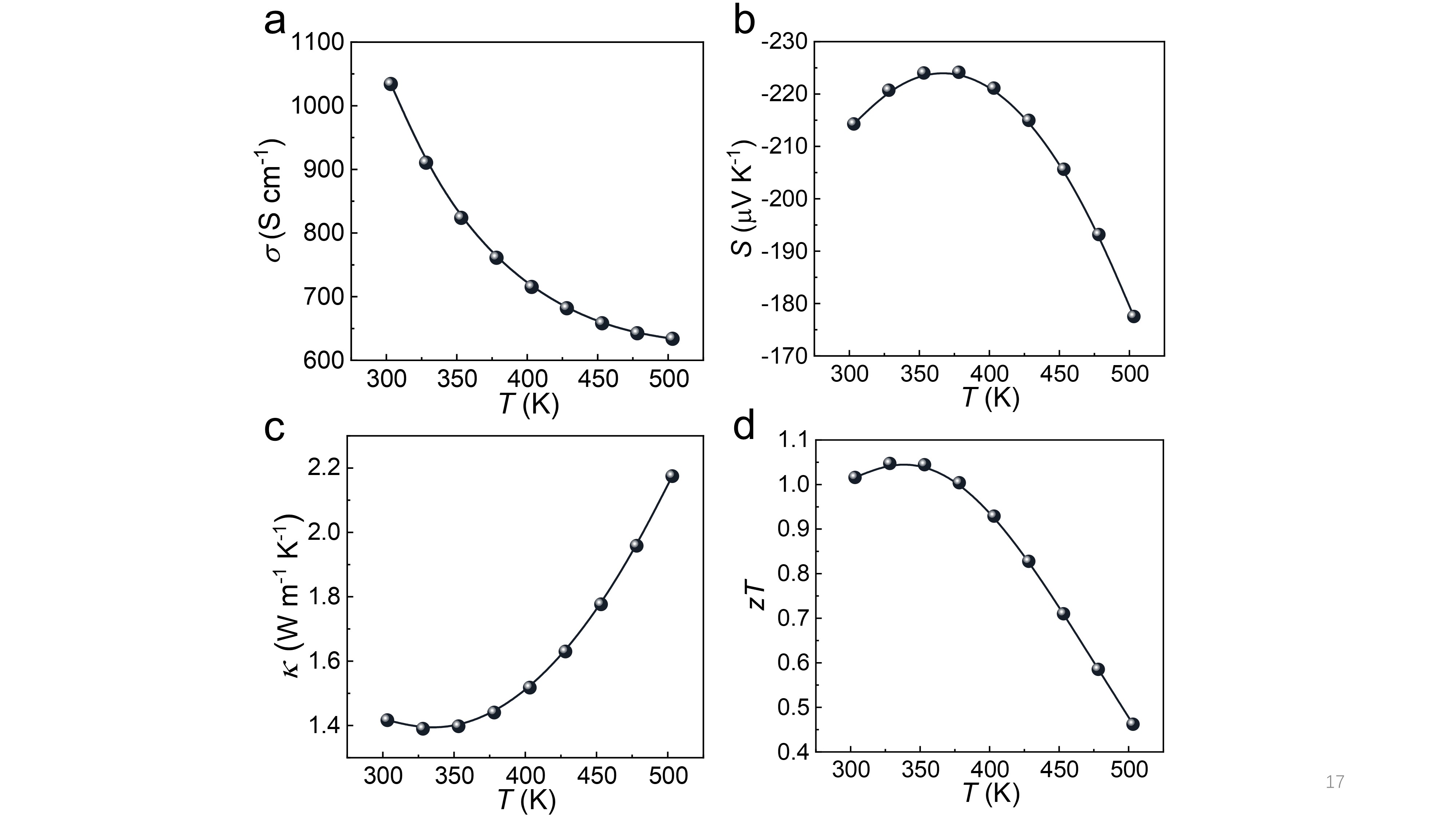


**Figure S13** Temperature-dependent (a) *σ*, (b) *S*, (c) *κ*, and (d) *zT* of the *n*-type Bi_2_Te_2.7_Se_0.3_.


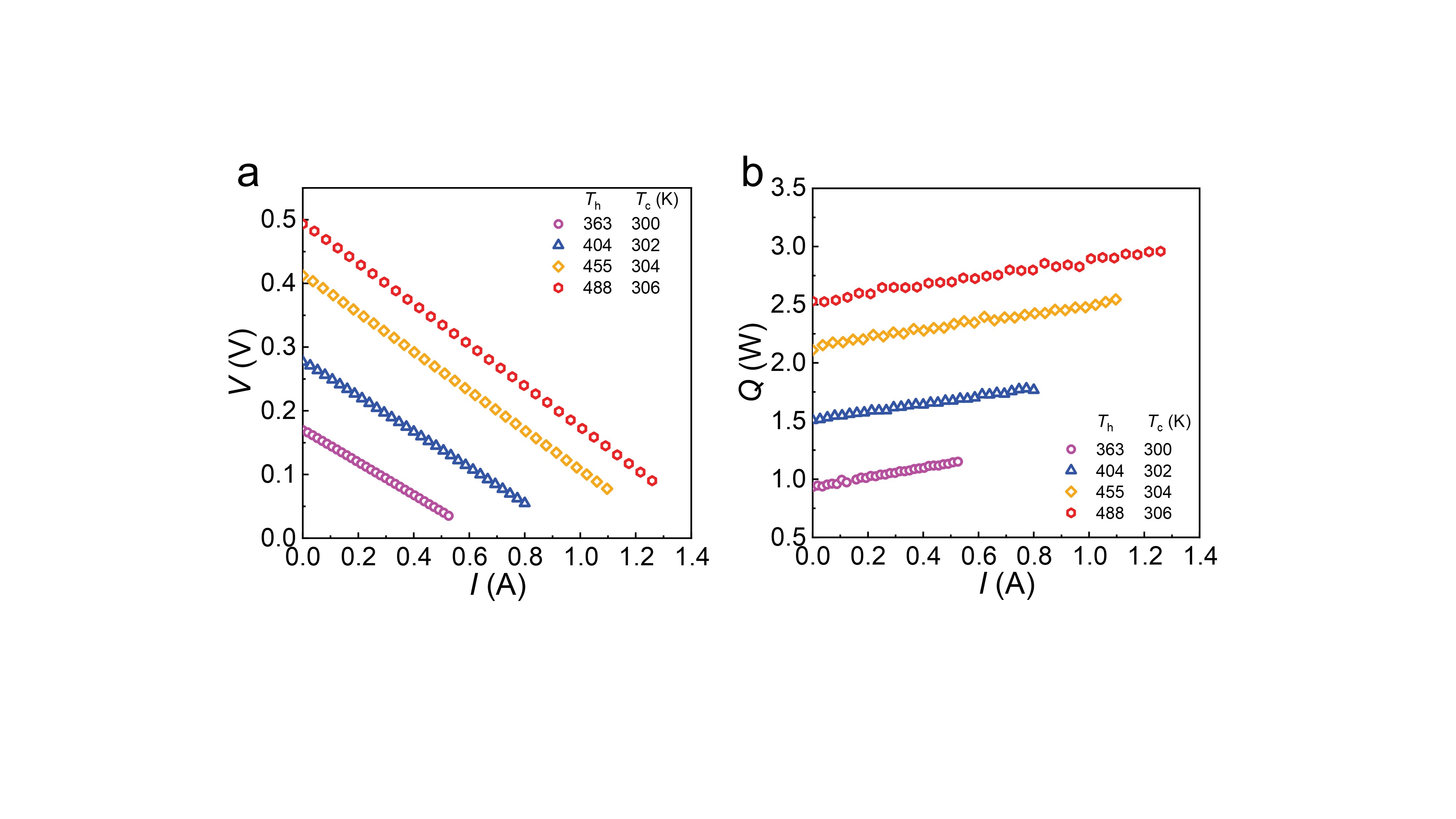


**Figure S14** (a) voltage (*V*) and (b) heat flow (*Q*) with current (*I*) at different temperature differences (Δ*T*) for the 7 pairs of modules prepared.


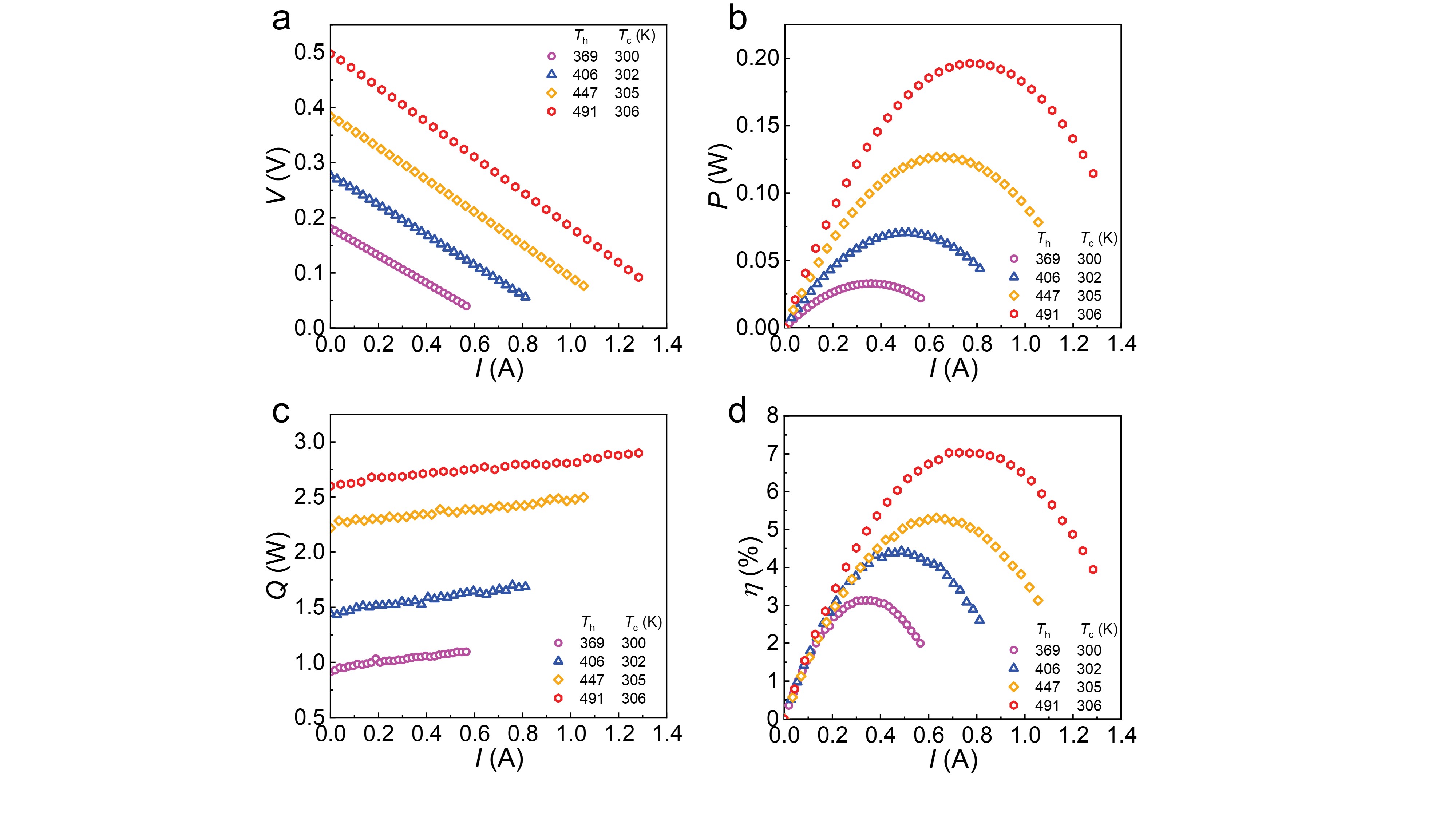


**Figure S15** Measurement data for Module 2. *I*-dependent (a) *V*, (b) output power (*P*), (c) *Q*, and (d) conversion efficiency (*η*) measured at different Δ*T*.


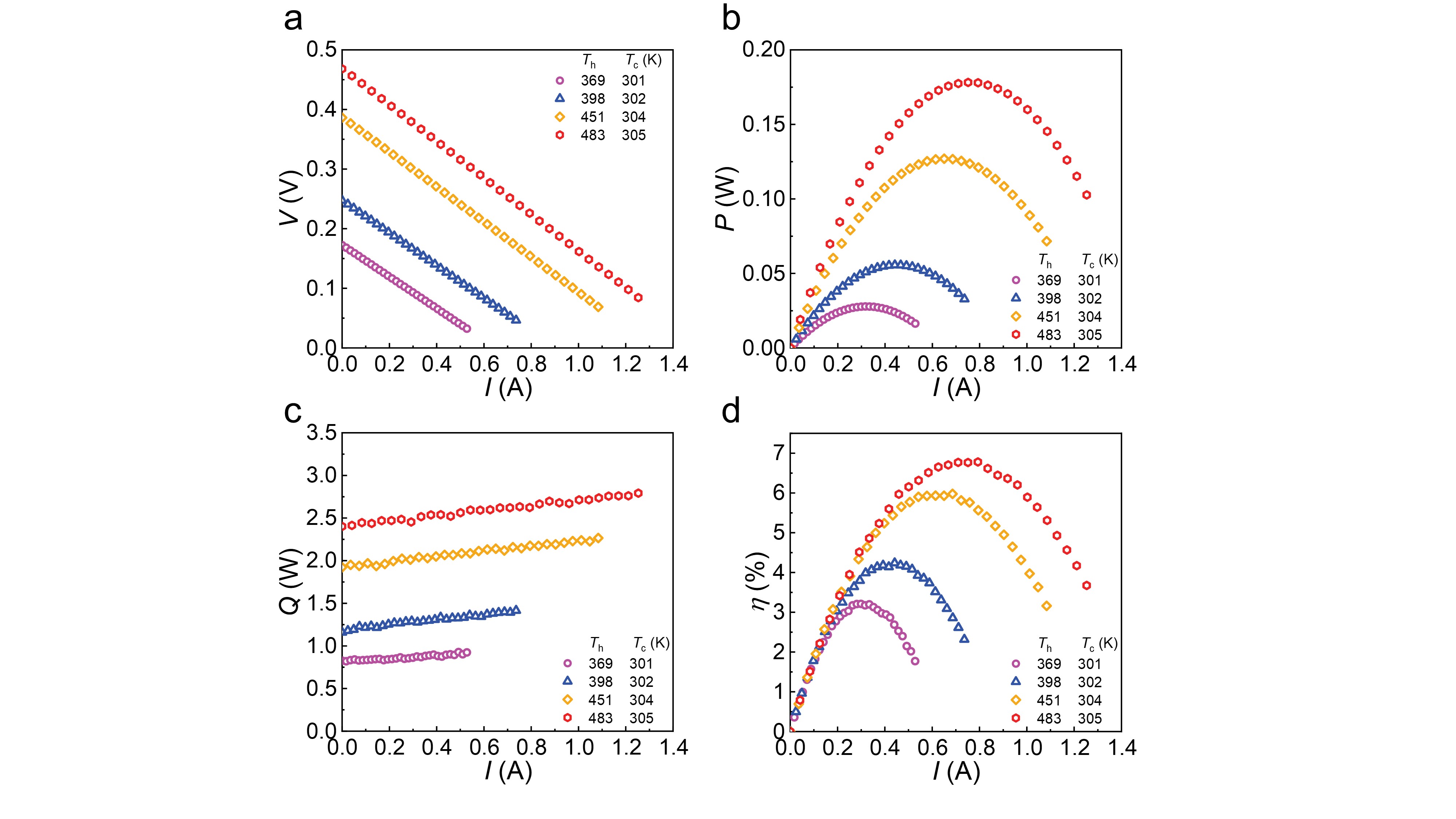


**Figure S16** Measurement data for Module 3. *I*-dependent (a) *V*, (b) *P*, (c) *Q*, and (d) *η* measured at different Δ*T*.

**Table S1** Density of Bi_0.4_Sb_1.6_Te_3.01_ + *x* wt.%Cu_3_SbSe_3_ samples in this work.

| **Composition** | **Measured density**  **(g cm^-3^)** | **Relative density**  **(%)** |
| --- | --- | --- |
| Bi_0.4_Sb_1.6_Te_3.01_ | 6.611 | 97.5 |
| Bi_0.4_Sb_1.6_Te_3.01_ + 0.03 wt.%Cu_3_SbSe_3_ | 6.603 | 97.4 |
| Bi_0.4_Sb_1.6_Te_3.01_ + 0.04 wt.%Cu_3_SbSe_3_ | 6.594 | 97.2 |
| Bi_0.4_Sb_1.6_Te_3.01_ + 0.05 wt.%Cu_3_SbSe_3_ | 6.590 | 97.2 |
| Bi_0.4_Sb_1.6_Te_3.01_ + 0.06 wt.%Cu_3_SbSe_3_ | 6.588 | 97.2 |

**Table S2** Fitting parameters for the samples using the expression of 𝜅_l_ = *aT*^–1^+ *b.*

|  | **Bi_0.4_Sb_1.6_Te_3.01_** | **Bi_0.4_Sb_1.6_Te_3.01_ + 0.04 wt.%Cu_3_SbSe_3_** |
| --- | --- | --- |
| *a* | 44.00455 | 49.96806 |
| *b* | 0.50166 | 0.40378 |

**Table S3** Parameters for the calculation of lattice thermal conductivity.

| Parameters | Description | Values | Ref. |
| --- | --- | --- | --- |
| $\theta_{D}$ | Debye temperature | 124 | Ref.^[14]^ |
| $v$ | Average sound velocity | 1922 m·s^-1^ | Exp. |
| $A_{N}$ | Comprehensive coefficient between  Umklapp and Normal processes | 2.3 | fitted |
| $\bar{V}$ | Average atomic volume of Bi_0.4_Sb_1.6_Te_3_ | 3.23×10^-29^ m^3^ | Ref.^[15]^ |
| $\bar{M}$ | Average atomic mass of Bi_0.4_Sb_1.6_Te_3_ | 2.2×10^-25^ kg | fitted |
| $\gamma$ | Grüneisen parameter | 1.47 | Exp. |
| $r$ | Poisson's ratio | 0.25 | Ref.^[14]^ |
| $v_{L}$ | Longitudinal sound velocity | 2979 m·s^-1^ | Exp. |
| $v_{T}$ | Transverse sound velocity | 1733 m·s^-1^ | Exp. |
| $\Gamma$ | Point defect scattering parameter | 0.175 | Ref.^[14]^ |
| d | Grain size | 2.15×10^-6^ m | Exp. |
| *N*_s_ | Number of stacking faults | 2.7×10^-6^ m^-1^ | Exp. |
| *N*_D_ | Dislocation density | 3.3×10^10^ cm^-2^ | Exp. |
| *B*_D_ | Magnitude of Burger's vector | 12.7 Å | Ref.^[14]^ |

**References**

[1] V. D. Blank, S. G. Buga, V. A. Kulbachinskii, V. G. Kytin, V. V. Medvedev, M. Y. Popov, P. B. Stepanov, V. F. Skok, *Phys. Rev. B* **2012**, *86*, 075426.

[2] G. Kresse, J. Furthmüller, *Phys. Rev. B* **1996**, *54*, 11169.

[3] J. Furthmüller, J. Hafner, G. Kresse, *Phys. Rev. B* **1996**, *53*, 7334.

[4] G. Kresse, J. Furthmüller, *Comp. Mater. Sci.* **1996**, *6*, 15.

[5] V. Wang, N. Xu, J.-C. Liu, G. Tang, W.-T. Geng, *Comput. Phys. Commun.* **2021**, *267*, 108033.

[6] J. P. Perdew, K. Burke, M. Ernzerhof, *Phys. Rev. Lett.* **1996**, *77*, 3865.

[7] Y. Pan, U. Aydemir, J. A. Grovogui, I. T. Witting, R. Hanus, Y. Xu, J. Wu, C.-F. Wu, F.-H. Sun, H.-L. Zhuang, J.-F. Dong, J.-F. Li, V. P. Dravid, G. J. Snyder, *Adv. Mater.* **2018**, *30*, 1802016.

[8] G. J. Snyder, A. H. Snyder, M. Wood, R. Gurunathan, B. H. Snyder, C. Niu, *Adv. Mater.* **2020**, *32*, 2001537.

[9] S. I. Kim, K. H. Lee, H. A. Mun, H. S. Kim, S. W. Hwang, J. W. Roh, D. J. Yang, W. H. Shin, X. S. Li, Y. H. Lee, *Science* **2015**, *348*, 109.

[10] T. J. Zhu, C. G. Fu, H. H. Xie, Y. T. Liu, B. Feng, J. Xie, X. B. Zhao, *Europhys. Lett.* **2013**, *104*, 46003.

[11] B. K. Singh, V. J. Menon, K. C. Sood, *Phys. Rev. B* **2006**, *74*, 184302.

[12] J. Callaway, H. C. von Baeyer, *Phys. Rev.* **1960**, *120*, 1149.

[13] B. Abeles, *Phys. Rev.* **1963**, *131*, 1906.

[14] H.-L. Zhuang, H. Hu, J. Pei, B. Su, J.-W. Li, Y. Jiang, Z. Han, J.-F. Li, *Energy Environ. Sci.* **2022**, *15*, 2039.

[15] Q. Zhang, M. Yuan, K. Pang, Y. Zhang, R. Wang, X. Tan, G. Wu, H. Hu, J. Wu, P. Sun, G.-Q. Liu, J. Jiang, *Adv. Mater.* **2023**, *35*, 2300338.
